# Metabolomics Profiling of Vitamin D Status in Relation to Dyslipidemia

**DOI:** 10.3390/metabo12080771

**Published:** 2022-08-22

**Authors:** Hanaa Mousa, Mohamed A. Elrayess, Ilhame Diboun, Simon K. Jackson, Susu M. Zughaier

**Affiliations:** 1 College of Medicine, QU Health, Qatar University, Doha P.O. Box 2713, Qatar; 2 Biomedical Research Center (BRC), QU Health, Qatar University, Doha P.O. Box 2731, Qatar; 3 Department of Human Genetics, Sidra Medicine, Doha P.O. Box 26999, Qatar; 4 School of Biomedical Sciences, Faculty of Health, University of Plymouth, Plymouth PL4 8AA, UK

**Keywords:** vitamin D, 25-hydroxyvitamin D, dyslipidemia, metabolomics, lipidomics

## Abstract

Vitamin D deficiency is a global disorder associated with several chronic illnesses including dyslipidemia and metabolic syndrome. The impact of this association with both dyslipidemia and vitamin D deficiency on metabolomics profile is not yet fully understood. This study analyses the metabolomics and lipidomic signatures in relation to vitamin D status and dyslipidemia. Metabolomics data were collected from Qatar Biobank database and categorized into four groups based on vitamin D and dyslipidemia status. Metabolomics multivariate analysis was performed using the orthogonal partial least square discriminate analysis (OPLS-DA) whilst linear models were used to assess the per-metabolite association with each of the four dyslipidemia/vitamin D combination groups. Our results indicate a high prevalence of vitamin D deficiency among the younger age group, while dyslipidemia was more prominent in the older group. A significant alteration of metabolomics profile was observed among the dyslipidemic and vitamin D deficient individuals in comparison with control groups. These modifications reflected changes in some key pathways including ceramides, diacylglycerols, hemosylceramides, lysophospholipids, phosphatidylcholines, phosphatidylethanol amines, and sphingomyelins. Vitamin D deficiency and dyslipidemia have a deep impact on sphingomyelins profile. The modifications were noted at the level of ceramides and are likely to propagate through downstream pathways.

## 1. Introduction

Vitamin D deficiency (serum 25 dihydroxy vitamin D (25(OH)D) concentrations <12 ng/mL) is a worldwide health problem affecting approximately 1 billion individuals globally, with vitamin D insufficiency (<20 ng/mL) affecting 50% of the population. The elderly, obese individuals, nursing home residents, and hospitalized patients have the greatest rates of vitamin D deficiency [1,2]. In Qatar, Al-Dabhani et al. found that 64% of the 1205 individuals in their research cohort were vitamin D deficient and suffered vitamin-D-related morbidity [3].

In recent years, a growing body of epidemiological and experimental data has shown that low blood vitamin D levels are associated with a variety of metabolic illnesses, including dyslipidemia, obesity, type 2 diabetes, insulin resistance, and cardiovascular disease, including hypertension [4]. In a study by Jiang et al., it was revealed that low vitamin D levels were inversely associated with LDL and triglycerides levels, whereas higher vitamin D levels were linked to high HDL [5]. HDL is dubbed as the “good” cholesterol, tasked with extracting excess cholesterol from peripheral arteries and transferring it to the liver for elimination in a process known as reverse cholesterol transfer [6]. As a result, guidelines recommend lowering triglyceride and LDL levels while increasing HDL levels. The activities of dyslipidemia medications, which reduce triglyceride and LDL levels while boosting HDL levels, support these guidelines.

Metabolic profiling has become an essential approach for identifying steady-state metabolite concentrations and researching metabolic system control [7,8]. Metabolomics and lipidomics studies assessing vitamin D in chronic diseases such as multiple sclerosis [9,10], inflammatory bowel disease (IBD) [11], cardiovascular diseases (CVD) [12], and diabetes revealed significant changes in specific metabolites [12,13]. Vitamin D supplementation influenced the metabolomics profile of overweight/obese African Americans in a dose-dependent manner. The study reported significant changes in ceramides and sphingomyelins, specifically increased levels of N-stearoyl-sphingosine (d18:1/18:0) (C18Cer) and stearoyl sphingomyelin (d18:1/18:0) (C18SM) [14]. Changes in ceramides such as *N*-palmitoyl-sphingosine and levels of sphingomyelins such as sphingosine-1-phosphate have been associated with vitamin D deficiency [14]. Sphingolipids including ceramides and sphingomyelin are a group of lipids that play a role in cell membrane integrity, cellular stress, and inflammatory signaling. Ceramides are the source of sphingomyelin. Sphingosine-1-phosphate (S1P) glycosphingolipids and sphingomyelin are two physiologically active sphingolipids that are formed from the latter [15,16].

The metabolic pathways linked with vitamin D insufficiency have been emphasized in the literature [17,18]. However, the metabolic signature of vitamin D sufficiency and deficiency in the context of dyslipidemia has not been investigated. In order to decipher the interplay between vitamin D status and dyslipidemia, this study profiled metabolic changes in four different categories of participants: vitamin D sufficient and normolipidemic (Group 1); vitamin D sufficient and dyslipidemia (Group 2); vitamin D deficient and dyslipidemia (Group 3); vitamin D deficient and normolipidemic (Group 4).

## 2. Materials and Methods

### 2.1. Study Design

This cohort is a cross-sectional, retrospective study based on data from 277 participants selected randomly from a larger cohort of 1820 subjects. The participants in the original cohort selected based on vitamin D level and dyslipidemia status. Those data were collected by the Qatar Biobank (QBB) [19,20]. This study was performed in line with the World Medical Association Declaration of Helsinki–Ethical Principles for medical research involving human subjects. The Institutional Research Board of Qatar University QU-IRB form (1366-E/20), QBB-IRB form (EX-2020-QBB-RES-ACC-0237-0124), approved all protocols. All participants consented to the use of their samples for research. Vitamin D status was dichotomized according to serum 25 dihydroxy vitamin D (25(OH)D) concentrations into a deficient <12 ng/mL and a sufficient ≥ 20 ng/mL. This classification was based on the Institute of Medicine’s recommendations [21]. Dyslipidemia was defined if any of the following cutoffs have been met: high total cholesterol (>6.2 mmol/L), high LDL-C (>4.1 mmol/L), and high TG (>2.3 mmol/L) [22].

The inclusion criteria were Qatari and non-Qatari healthy adults. The exclusion criteria included those using vitamin D supplements, pregnancy, and those with chronic diseases such as diabetes, high blood pressure, asthma, hay fever, blood clot, heart attack, angina, stroke, emphysema/chronic bronchitis, hyperthyroidism hyperparathyroidism, Cushing syndrome, and cancer. The 277 participants were further divided into four groups according to the serum 25 (OH)D concentrations and dyslipidemia presence: vitamin D sufficient and normolipidemic (Group 1, n = 64); vitamin D sufficient and dyslipidemia (Group 2, n = 26); vitamin D deficient and dyslipidemia (Group 3, n = 88); vitamin D deficient and normolipidemic (Group 4, n = 99).

### 2.2. Physical and Biochemical Measurements

Participants’ height and weight were measured by trained nurses. Bodyweight was measured using the TANITA BC-418 MA instrument. BMI was calculated by dividing weight in kg by height in m2. Waste to hip ratio (WHR) was calculated by dividing the waist by cm on the hip. Venous blood samples were collected from participants after overnight fasting. For biochemical measurements, blood specimens were sent to Hamad Medical Corporation Laboratories (College of American Pathologist Accredited Laboratory) for analysis. White blood cells (WBC), monocytes, lymphocytes, neutrophils, and eosinophils were measured as part of the differential white blood cell count. Serum 25(OH)D concentration (included both vitamin D2 and vitamin D3 fractions) was measured using electrochemiluminescence immunoassay (LIAISON^®^ 25-hydroxyvitamin D Total Assay, DiaSorin Inc., Stillwater, MN, USA). Serum total cholesterol was measured with the enzymatic CHOD-PAP method. HDL cholesterol Plus Third Generation Method was used to measure the serum HDL cholesterol. LDL cholesterol Plus Second Generation Method was used to measure the serum LDL cholesterol. Serum triacylglycerol was measured with the enzymatic GPO-PAP method. Plasma glucose was measured with the enzymatic/amperometric method (Nova stat strip and Roche Accucheck Inform II devices). C peptide, HBA1C, and insulin were measured using the immunoassay method. Technical methodology details are previously described [19,20].

### 2.3. Metabolomics and Lipidomic Profiling

In this study, 1158 metabolites that were annotated or previously identified by Metabolon were used in this study. The established procedures were applied for untargeted metabolomics of serum samples from all individuals as previously described [23]. Briefly, metabolite measurements were performed using Waters ACQUITY ultra-performance liquid chromatography (UPLC) and a Thermo Scientific Q-Exactive high resolution/accurate mass spectrometer interfaced with a heated electrospray ionization (HESI-II) source and an Orbitrap mass analyzer with a 35,000 mass resolution. In short, methanol was used to extract serum samples to eliminate the protein fractions. Subsequently, the extracts were divided into five fractions: two for analysis by two separate reverse phase (RP)/UPLC-MS/MS methods with positive ion mode electrospray ionization (ESI), one for analysis by RP/UPLC-MS/MS with negative ion mode ESI, one for analysis by hydrophilic interaction chromatography (HILIC)/UPLC-MS/MS with negative ion mode ESI, and one sample was reserved for backup. Later, the peaked were identified using Metabolon’s hardware. Peaks were matched to existing library entries of pure standards of over 3300 pure standard chemicals to identify compounds. Compounds were then classified into distinct groups based on their origins, as previously discussed [24].

Statistical analyses were carried out using IBM SPSS version 25, R version 3.6 (Armonk, New York, NY, USA), and SIMCA 16.0.1 software (Umetrics, Umeå, Sweden). Baseline characteristics of four groups were presented in mean and (SD) for continuous variables. For skewed variables, the median was calculated. ANOVA was used for biomarkers; age, BMI, waist-to-hip ratio, white blood cell, monocyte, lymphocyte, neutrophil, eosinophil, C Peptide, total cholesterol, LDL, HDL-c, Triglyceride, HBA1C, glucose, insulin, and monocyte percentage to HDL-c ratio (MHR). *p*-value *p* < 0.05 was considered statistically significant.

A linear model was fit per metabolite (y-variable) that incorporated the explanatory categorical variable study group (featuring four levels Group 1, Group 2, Group 3 and Group 4) as well as the following confounders: BMI, age, the first two PCs from PCA analysis and gender. R package Emmeans was used to extract the desired pairwise contrasts. The *p* values were penalized for multiple testing using the FDR procedure. Enrichment analysis of pathway/structural classes of metabolites was performed on the list of metabolites with nominal *p* value less than or equal to 0.05 from each contrast based on the Fishers’ exact test, followed by FDR multiple testing correction.

## 3. Results

### 3.1. General Characteristics of Participants

The total number of participants was 277 including 38.9% female and 61.1% male participants. The cohort was divided into four groups based on vitamin D status and serum lipids profile. Group 1 was composed of 64 participants who were vitamin D sufficient (greater than 20 ng/mL) with normolipidemia, hence considered as the control group. Group 2 was composed of 26 participants who were vitamin D sufficient with dyslipidemia. Group 3 was composed of 88 participants who were vitamin D deficient (less than 12 ng/mL) with dyslipidemia. Group 4 was composed of 99 participants who exhibited vitamin D deficiency with normolipidemia. Dyslipidemia was more prominent in the older age group, although they were vitamin D sufficient, as opposed to the younger age group with a higher prevalence of vitamin D deficiency. Higher BMI was observed in dyslipidemia groups in comparison with normolipidemic groups. Moreover, the inflammation marker monocyte to HDL ratio (MHR) was significantly higher in dyslipidemic groups (Group 2 and Group 3) in comparison with normolipidemic participants. The median for the glycemic variables C Peptide has the highest value 2.76 (ng/mL) in Group 3 of participants with combined vitamin D deficiency and dyslipidemia. Additionally, HDL has the lowest value in this group, although it was low in both dyslipidemic and vitamin D deficiency groups. Of note, triglycerides levels among participants with combined vitamin D deficiency and dyslipidemia were almost double that of other groups. There were differences in the distribution of gender in the four studied groups. For instance, males were more dominant in Group 3 (dyslipidemia and vitamin D deficiency) than females, whereas females were more dominant in Group 4 (normolipidemia and vitamin D deficiency) (Table 1).

**Figure 1:**
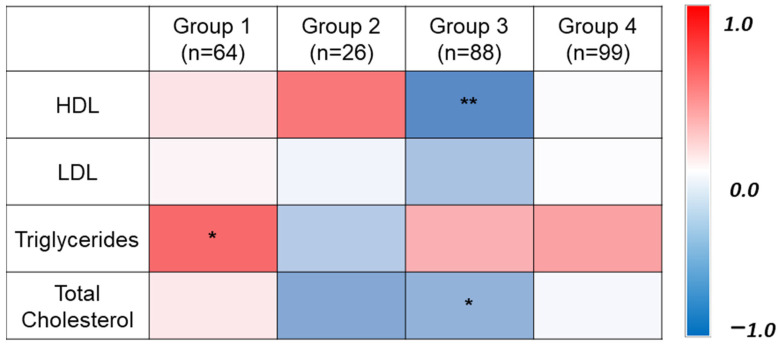
The heat map illustrates Pearson correlation depicting the association between lipid profile components (HDL, LDL, Triglycerides, and Total cholesterol) and serum 25(OH)D among four groups. * *p* ≤ 0.05, ** *p* ≤ 0.01. Group 1 (Vitamin D sufficient and normolipidemic); Group 2 (Vitamin D sufficient and dyslipidemia); Group 3 (Vitamin D deficient and dyslipidemia); Group 4 (Vitamin D deficient and normolipidemic).

Using Pearson correlation, a strong negative association has been detected between HDL and vitamin D level in Group 3 (Vitamin D deficient and dyslipidemia) (Figure 1).

### 3.2. Metabolites and Lipids Analysis

**Figure 2:**
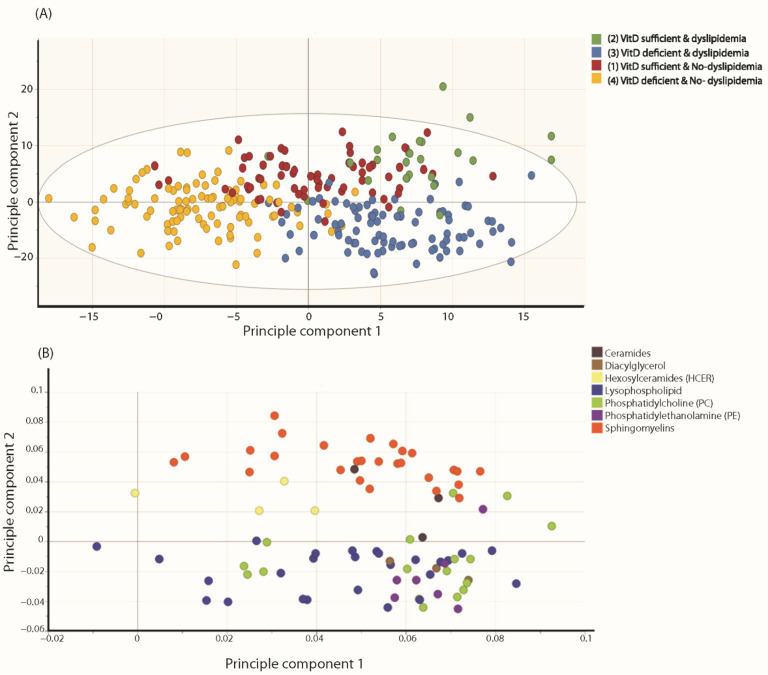
Illustration of OPLS-DA analysis for the four groups. (**A**): represents a scatter plot of two principal components, distinguishing all four groups in a two-dimensional space. (**B**): represents a loading plot highlighting the weights of the metabolites in all four groups, indicating top metabolite responsible for group separation. R2Y (cum) was 0.637, and Q2 (cum) was 0.229. Vitamin D sufficient and normolipidemic (Group 1, n = 64); Vitamin D sufficient and dyslipidemia (Group 2, n = 26); Vitamin D deficient and dyslipidemia (Group 3, n = 88); Vitamin D deficient and normolipidemic (Group 4, n = 99).

The orthogonal partial least square discriminate analysis (OPLS-DA) of metabolites and lipids associated with vitamin D status stratified by dyslipidemia levels revealed two significant class-discriminatory components accounting for 95% of the variation in the data due to group membership. R2Y (cum) was 0.637, and Q2 (cum) was 0.229. Figure 2A shows a scatter plot of these two components, distinguishing all four groups in a two-dimensional space representation. Interestingly, the first predictive component (x-axis) projects the dyslipidemia status whilst the second predictive component is an indicator of vitamin D levels. (Figure 2A). The relative abundance of metabolite enrichment is important, and thus, the variable influence on projection (VIP) list indicating top metabolites that differentiate the four groups is shown in Appendix S1. The loading plot (Figure 2B) indicates the weights of the metabolites underlying the separation of subjects in the score plot in Figure 2A. The metabolic pathways that appeared confined to certain groups and not others are highlighted in color. These include ceramides, diacylglycerols, hemosylceramides, lysophospholipids, phosphatidylcholines, phosphatidylethanol amines and sphingomyelins (Figure 2B). Cholesterol (as a single metabolite) was assigned a high weight by the OPLS-DA, which indicates a high discriminatory capacity for group three; representing vitamin D deficient and dyslipidemic subjects. In addition, sphingomyelins/lysophospholipids are markers of vitamin D sufficiency and deficiency, respectively. Moreover, we noted an enrichment in ceramides, phosphatidylcholine (PC), phosphatidylethanolamine (PE), and diacylglycerol in the dyslipidemic group as opposed to a marked presence of hexosylceramides in the normolipidemics controls.

#### Metabolomics and Lipidomic Signatures in the Participants’ Groups

Linear regression analysis was used to find an association between group pairs, the cut-off value of significance for FDR, and the *p*-value was (≤0.05). Examples of Metabolomics signatures are shown using volcano plots in Supplementary Figure S1.

**Figure 3:**
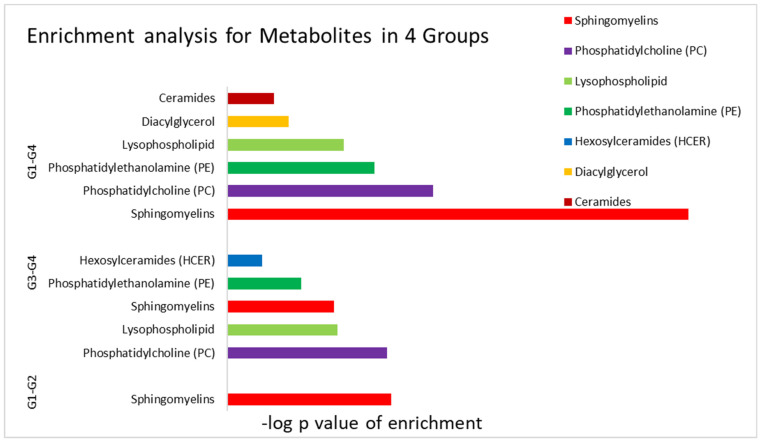
Illustration of the enriched pathway in paired group contrasts. A significant alteration was prominent among sphingomyelins pathway components. Vitamin D sufficient and normolipidemic (Group 1, n = 64); vitamin D sufficient and dyslipidemia (Group 2, n = 26); vitamin D deficient and dyslipidemia (Group 3, n = 88); vitamin D deficient and normolipidemic (Group 4, n = 99).

In vitamin D sufficient groups (Group 2 versus Group 1) (dyslipidemic versus normolipidemic participants), univariate analysis revealed twenty metabolites that were associated with dyslipidemia among vitamin D sufficient participants—namely cholesterol, 1-palmitoyl-2-docosahexaenoyl-GPE, 1-oleoyl-2-docosahexaenoyl-GPC, CMPF, and various sphingomyelins metabolites, which were significantly more abundant in the dyslipidemia group (Supplementary Table S1). Enrichment analysis revealed a marked upregulation of sphingomyelins in the dyslipidemia group at physiological levels of vitamin D (Table 2 and Figure 3).

In vitamin D deficient groups (Group 4 versus Group 3) (Normolipidemic versus dyslipidemic participants), a wider range of complex lipids was found to plummet in the dyslipidemic group. Univariate analysis also highlighted other metabolites including oleoyl-linoleoyl-glycerol, retinol, cholesterol, alpha-tocopherol, and amino acids leucine, isoleucine, and valine were also found to differ in their levels in the dyslipidemic group with deficient vitamin D in comparison to their corresponding controls (Supplementary Table S2). Enrichment analysis revealed a marked upregulation of sphingolipids, fatty acids, phosphatidylcholines (PCs), phosphatidylethanolamines (PEs), lysophospholipid and ceramides (Table 2 and Figure 3).

In normolipidemics groups (Group 4 versus Group 1) of vitamin D deficient versus sufficient participants, eleven metabolites were significantly associated with vitamin D deficient participants without dyslipidemia, i.e., Group 4 (Supplementary Table S3). Metabolites such as 1-oleoyl-2-docosahexaenoyl-GPC, ergothioneine, urea, valine, 4-methyl-2-oxopentanoate, arabo.te/xylo.te, 1-(1-enyl-oleoyl)-GPE (*p*-18:1), allantoin, methionine sulfone, 1-(1-enyl-stearoyl)-GPE (*p*-18:0), tryptophan), and most notably ergothioneine is higher in vitamin D sufficiency compared to deficiency (Supplementary Table S3). Enrichment analysis delineated the following significant categories: sphingomyelins, phosphatidylcholine (PC), phosphatidylethanolamine (PE), lysophospholipid, diacylglycerol, and ceramides (Table 2 and Figure 3).

In dyslipidemics groups (group 3 versus group 2) of vitamin D deficient versus sufficient participants, univariate analysis was significant for 15 metabolites, including hydroxy-CMPF and CMPF, which were significantly reduced in vitamin D deficient Group 3 (Supplementary Table S4). Other metabolites such as (docosahexaenoic (DHA; 22:6n3), S-methyl cysteine sulfoxide, 1-oleoyl-2-docosahexaenoyl-GPC, perfluorooctanoate (PFOA), tartro.te (hydroxymalo.te), 2-hydroxyglutarate, lysine, and sphingomyelin pathway followed the same pattern of regulation (Supplementary Table S4). The univariate analysis for other groups, e.g., Group 1 (vitamin D sufficient versus normolipidemic) and Group 3 (vitamin D deficient and dyslipidemia) didn’t reveal any significance difference in metabolites enrichment.

### 3.3. Quantitative Measurement of Single Specific Metabolites Distribution among Participants

**Figure 4:**
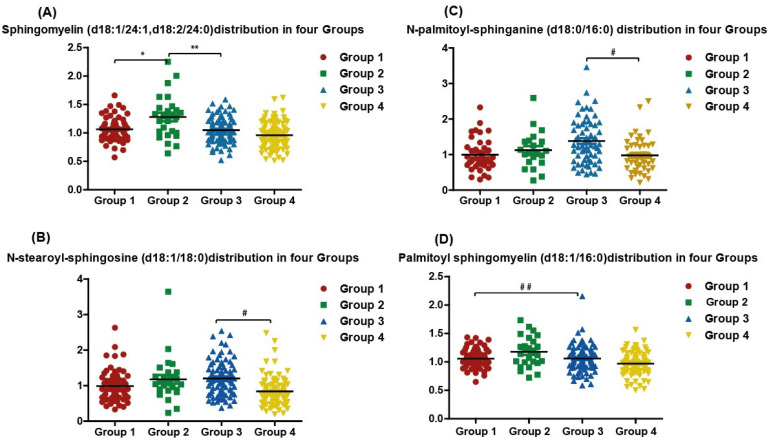
Metabolites quantity distribution among four groups of participants. (**A**) sphingomyelin (d18:1/24:1,d18:2/24:0); (**B**) N-stearoyl-sphingosine (d18:1/18:0); (**C**) N-palmitoyl-sphingosine (d18:0/16:0); and (**D**) palmitoyl sphingomyelin (d18:1/16:0). Vitamin D sufficient and normolipidemic (Group 1, n = 64); vitamin D sufficient and dyslipidemia (Group 2, n = 26); vitamin D deficient and dyslipidemia (Group 3, n = 88); vitamin D deficient and normolipidemic (Group 4, n = 99). Y axis is log transformed abundance levels of metabolites. * Significant *p* value comparing Group 1 to Group 2. ** Significant *p* value comparing Group 2 to Group 3. # Significant *p* value Group 3 to Group 4. # # Significant *p* value comparing Group 1 to Group 3.

Sphingomyelin showed the highest value in the dyslipidemic vitamin D sufficient group, i.e., Group 2 (Figure 4A), whereas, N-stearoyl-sphingosine (d18:1/18:0) and N-palmitoyl-sphingosine (d18:0/16:0) were higher in the dyslipidemic and vitamin D deficient group individuals, i.e., Group 3 (Figure 4B,C). When comparing vitamin D sufficient and normolipidemic, i.e., Group 1, with vitamin D deficient and dyslipidemic, i.e., Group 3, Palmitoyl sphingomyelin (d18:1/16:0) was significantly elevated in the latter group 3 (Figure 4D).

**Figure 5:**
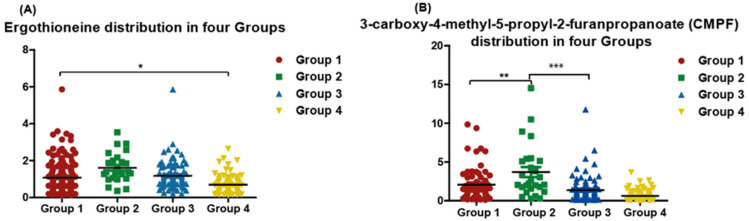
Quantitative distribution of Ergothioneine (**A**) and 3-carboxy-4-methyl-5-propyl-2-furanpropanoate (CMPF) (**B**) among four groups. Vitamin D sufficient and normolipidemic (Group 1, n = 64); vitamin D sufficient and dyslipidemia (Group 2, n = 26); vitamin D deficient and dyslipidemia (Group 3, n = 88); vitamin D deficient and normolipidemic (Group 4, n = 99). Y axis is log transformed abundance levels of metabolites. * Significant *p* value comparing Ergothioneine levels in Group 1 (Vitamin D sufficient) to Group 4 (Vitamin D deficient). ** Significant *p* value comparing CMPF levels in Group 1 (normolipidemic) to Group 2 (dyslipidemic). *** Significant *p* value comparing CMPF levels in Group 2 (Vitamin D sufficient) to Group 3 Vitamin D deficient in dyslipidemic participants.

Interestingly, the amino acid ergothioneine was reduced in vitamin D deficient normolipidemic in comparison to vitamin sufficient participants (Figure 5A). Upon comparison between the different groups, 3-carboxy-4-methyl-5-propyl-2-furanpropanoate (CMPF) level was higher in vitamin D deficient and dyslipidemic, i.e., Group 3 (Figure 5B).

## 4. Discussion

Worldwide, vitamin D insufficiency and dyslipidemia are common [25,26]. Vitamin D is well-known for its anti-inflammatory properties. This vitamin deficit may result in and be associated with a variety of illnesses [27]. The disruption in the lipids profile that occurs in dyslipidemia is also associated with various chronic conditions, most notably metabolic syndrome and cardiovascular diseases [28]. Individuals featuring combined vitamin D insufficiency and dyslipidemia are predisposed to a variety of chronic diseases. However, the metabolomics signature for this group of individuals has not yet been thoroughly investigated. In this work, we examined the effect of vitamin D status on metabolomics signatures of participants with and without dyslipidemia both separately and combined. 

The findings of this study revealed that dyslipidemia was more prevalent in the older age group, despite vitamin D sufficiency. Vitamin D deficiency, on the other hand, was more common among the young age group. Sphingomyelins were more notably altered in vitamin D deficiency without dyslipidemia as the sphingomyelin pathway was disrupted. Phosphatidylcholine (PC) and phosphatidylethanolamine (PE) were found to be highly altered among vitamin D deficient participants with and without dyslipidemia. Ergothienone levels were substantially higher in vitamin D adequate people compared to vitamin D deficient persons. Vitamin D is synthesized from the main precursor 7-dehydrocholesterol present in the skin, thereby it may be affected by dyslipedmia status. Several studies and meta-analysis reported the associations between low vitamin D status and altered lipid profile, i.e., dyslipidemia [29,30]. Further, vitamin D possesses immune modulatory effects that impact inflammation status and consequently lipid homeostasis including HDL-c biogenesis. Mousa et al. [31] observed the inverse association between vitamin D status and the subclinical inflammation marker monocyte percentage to HDL ratio (MHR) [31].

Sphingolipids, along with phosphoglycerides (also known as glycerophospholipids), are important components of cellular membranes, forming the two leaflets of the cellular membrane. The lipid bilayer structures protect the structure and stability of the cellular membrane. Phosphoglycerides include phosphatidylcholine (PC), phosphatidylethanolamine (PE), and phosphatidylserine (PS). Phosphoglycerides are made up of glycerol units and fatty acids. The inner cellular membrane leaflet is mostly composed of phosphatidylethanolamine that accounts for 25% of all phospholipids and is crucial for distributing charges and membrane stability as it contains amine group [32,33]. Sphingolipids are made up of a hydrophobic ceramide backbone and a lengthy hydrocarbon chain of amino alcohol. In mammalian cells, the primary sphingolipids are sphingomyelin (SM) and glycosphingolipids (GSLs) [34]. Sphingomyelins are key components of a nerve cell’s myelin sheath. Sphingomyelin dysfunction has been linked to a number of autoimmune disorders, most notably multiple sclerosis [35]. Phosphoglycerides and sphingolipids are lipid metabolites that have a tight relationship with lipoproteins, notably HDL. HDL’s primary lipid components are phosphatidylcholine (PC) and sphingomyelin (SM) [36]. However, increasing the quantity of sphingomyelin in HDL particles impairs HDL formation and maturation [37]. This study investigated the levels of sphingolipids and ceramides-related metabolites among the normolipidemic, dyslipidemic, vitamin sufficient and deficient individuals. N-stearoyl-sphingosine (d18:1/18:0), one of long chain ceramides, was noted higher in the dyslipidemic and vitamin D deficient group (group 3 population); rather contradicting with the findings by Chen et al. and Koch et al. Indeed, an increase in N-stearoyl-sphingosine (d18:1/18:0) upon the increase of vitamin D was noted after the administration of vitamin D3 supplementation in a dose and time-dependent manner [14,38]. The noted increase in ceramide despite the reduction in vitamin D levels could be due to the effects of dyslipidemia on the metabolomic profile [39].

The lysophospholipids category is made up of lipids that act as an intermediary in the creation of other lipids in cells. They are classified as either lysoglycerophospholipids or lysosphingolipids [40]. Our findings show an increase in lysophospholipids in the vitamin D deficient and dyslipidemic groups. Lysophospholipids are linked to several diseases such as asthma and chronic obstructive pulmonary [41]. One of the lysosphingolipids is sphingosine 1-phosphate (S1P), which is a bioactive sphingolipid that functions as a signaling molecule [42]. It regulates innate and adaptive immunity and is implicated in atherosclerosis, in addition to modulating numerous physiological and biological variables such as cell migration and proliferation [43,44]. The majority of S1P in the blood is associated with HDL [45]. Unlike in tissue, S1P is present in large amounts in the blood; however, it is rapidly degraded [46]. ApoM is an apolipoprotein that binds to S1P on HDL particles and has a significant influence on HDL biogenesis [47]. Vitamin D, on the other hand, has been shown to diminish the expression of S1P1, S1P2 receptors while increasing the expression of S1P3, S1P4 receptors [48,49]. As a result, lysosphingolipids (mostly S1P) have an indirect influence on dyslipidemia through modulating HDL synthesis. Simultaneously, vitamin D reduces the S1P action by targeting the S1P primary receptors.

In vivo studies linked ceramides to diabetes and insulin resistance. For instance, feeding mice with a high-fat diet resulted in an increase in long chain ceramides, leading eventually to obesity and insulin resistance [50,51]. N-palmitoyl-sphingosine (d18:1/16:0) (C16Cer) particularly was linked to cardiovascular disease and obesity-related insulin resistance [52,53]. In vitro investigations revealed that vitamin D has a direct effect on both phosphoglycerides and palmitoyl sphingomyelin (d18:1/16:0), stearic, linoleic, and arachidonic acid while increasing palmitic acid [54]. N-palmitoyl-sphingosine and N-stearoyl-sphingosine specifically were linked to cardiovascular mortality [52]. Treatment with vitamin D metabolites, on the other hand, activated the sphingomyelin pathway, resulting in a large rise in ceramide concentration and a proportionate reduction in sphingomyelin [55]. In this investigation, we report that in vitamin D deficient groups, various sphingomyelins with long acyl chain-like (C18, C17, and C24) acyl chain-like sphingomyelins were enriched in the dyslipidemia group compared to the normolipidemic group. This finding is consistent with that of Hanamatsu et al., who reported that serum sphingomyelins with saturated acyl chains (C18:0, C20:0, C22:0, and C24:0) are raised in obese people and may be linked to the development of metabolic syndrome [56]. In the vitamin D deficient and dyslipidemic group, we observed an increase in sphingomyelins with long acyl chain-like (C18, and C17), as well as an increase in ceramides and phosphoglyceride products such as phosphatidylcholine (PC) and phosphatidylethanolamine (PE). This might imply that patients with low serum vitamin D concentrations as well as dyslipidemia have an excess of both phosphoglycerides and sphingolipids.

Ergothioneine is an amino acid that is mostly generated by fungus and bacteria and is considered a potent antioxidant. It cannot be synthesized by the human body, but it can be obtained through dietary sources such as mushrooms and black beans [57]. Ergothioneine levels were shown to be low in the aged population, particularly those with cognitive impairment [58]. Ergothioneine levels have also been reported to be lower in several neurological illnesses, such as Parkinson’s [59]. Ergothioneine was reported to reduce oxidative stress and inflammatory indicators such as allantoin (urate oxidation), 8-hydroxy-2′-deoxyguanosine (responsible for DNA damage), 8-iso-prostaglandin F2 (responsible for excessive lipid peroxidation), and C-reactive protein [60]. In this study, ergothioneine levels in the vitamin D adequate group were higher than those in the deficient group with normolipidemic status. This might be due to dietary factors; for example, foods high in vitamin D also contain high levels of ergothioneine.

## 5. Conclusions

Vitamin D deficiency and dyslipidemia have a profound impact on several individual metabolites including CMPF and Ergothioneine, in addition to multiple pathways, particularly sphingmyelins and ceramides. The modifications were also noted on the downstream pathways including phosphatidylcholine (PC), and phosphatidylethanolamine (PE). This impact was highest among subjects with combined vitamin D deficiency and dyslipidemia.

### Limitation of the Study

The history of medication, particularly drugs which have an impact on calcium and phosphorus metabolism and self-prescribed lipid lowering agents, was missing. This could have influenced data, in addition to other unmeasured factors including environmental and behavioral factors, e.g., dietary habit. However, the linear model could report some confounders, helping to narrow the gap. An additional limitation is the lack of in vitro or in vivo studies to validate the result.

## Figures and Tables

**Table 1:** Characteristics of the participants’ groups based on serum 25(OH)D concentrations and dyslipidemia.

	Total	25(OH)D Sufficient, Normolipidemia (G1)	25(OH)D Sufficient, Dyslipidemia (G2)	25(OH)D Deficient, Dyslipidemia (G3)	25(OH)D Deficient Normolipidemia (G4)	
	n = 277	n = 64	n = 26	n = 88	n = 99	*p* Value
25(OH)D ng/ml	14.8 (8.3)	22 [20, 26] *	24.5 [22, 29.2] *	10 (1.8)	9.7 (1.8)	<0.001
Age	34.7 (10.8)	38.7 (11.04)	43.8 (8.9)	37.5 (9.2)	26 [21, 31] *	<0.001
Gender						
Males %	61.4	53.1	65.4	79.5	49	
BMI	27.6 (5.7)	26.8 (5.0)	27.9 (4.3)	29.4 (4.9)	26.5 (6.7)	0.002
HWR ^a^	0.8 (0.1)	0.8 (0.1)	0.9 (0.1)	0.9 (0.1)	0.8 (0.1)	<0.001
WBC x103/µl	6.5 (1.8)	6.2 (1.2)	5.8 [5.0, 6.4] *	6.98 (2.0)	6.4 (1.7)	0.006
Monocyte %	7.8 (2.1)	7.6 (1.87)	8.1 (1.6)	7.84 (1.8)	7.5 [6.3, 8.8] *	0.750
Neutrophil %	53.1 (8.9)	54.0 (8.5)	50.4 (9.7)	52.58 (8.9)	53.8 (8.6)	0.266
Lymphocyte %	35.5 (7.8)	34.9 (7.8)	37.3 (8.5)	35.87 (8.1)	35.1 (7.2)	0.533
Eosinophil %	3.0 (2.0)	2.6 [1.5, 3.6] *	3.7 [2.2, 5.0] *	2.75 [2.1, 3.7] *	2.3 [1.3, 3.5] *	0.044
MHR ^b^	6.7 (3.0)	5.6 [4.1, 7.22] *	7.01 (2.7)	7.2 [5.2, 8.7] *	5.7 [4.4, 7.5] *	0.001
Total Cholesterol (mmol/l)	4.9 (1.0)	4.7 (0.7)	5.9 (0.7)	5.6 (1.0)	4.2 (0.5)	<0.001
HDL-C (mmol/l)	1.29 (0.4)	1.4 (0.4)	1.25 (0.3)	1.11 [0.9, 1.3] *	1.3 (0.3)	<0.001
LDL (mmol/l)	2.96 (0.9)	2.8 (0.6)	3.79 (0.7)	3.5 (1.0)	2.4 (0.5)	<0.001
Triglycerides (mmol/l)	1.48 (1.1)	1.1 (0.4)	1.55 [1.1, 2.5] *	2.2 [1.2, 2.7] *	1.0 (0.3)	<0.001
HBA.1C %	5.3 (0.5)	5.4 [5.1, 5.5] *	5.49 (0.3)	5.4 (0.7)	5.2 (0.3)	0.015
Glucose (mmol/L)	5.14 (0.9)	5 [4.5, 5.4] *	5 [4.9, 5.6] *	5.1 [4.7, 5.6] *	4.9 (0.5)	0.004
Insulin (µU/mL)	18.4 (26)	7.9 [5, 12.9] *	9.2 [7.0, 19.4] *	14.2 [8.2, 29.8] *	9.6 [6.0, 13.0] *	<0.001
C. Peptide (ng/mL)	3.0 (2.3)	2.1 [1.4, 2.7] *	2.1 [1.7, 4.0] *	2.76 [2.1, 4.9] *	1.97 [1.5, 2.9] *	<0.001

Data is represented as mean (SD) or median (IQR) for skewed data. *p* value was in reference to control group (1) (Vitamin D sufficient, and Normolipidemic). ^a^ Hip-to-waist ratio ^b^ MHR Monocyte percentage to HDL Ratio * Represent Median [Interquartile range]

**Table 2:** Metabolites enrichment among various groups based on vitamin D status with or without dyslipidemia.

Pathway	*p* Value	FDR
Metabolites enriched in vitamin D sufficient groups; Normolipidemic versus vitamin D sufficient dyslipidemic participants (group 2 versus group 1)		
Sphingomyelins	1.97 × 10^−13^	<0.001
Metabolites enriched in different levels in vitamin D deficient Normolipidemic versus vitamin D sufficient dyslipidemic participants (Group 4 versus Group 3)		
Phosphatidylcholine (PC)	4.42 × 10^−13^	<0.001
Lysophospholipid	2.87 × 10^−9^	<0.001
Sphingomyelins	5.46 × 10^−9^	<0.001
Phosphatidylethanolamine (PE)	1.77 × 10^−6^	<0.001
Hexosylceramides (HCER)	1.87 × 10^−3^	0.032
Metabolites enriched in different levels in normolipidemics vitamin D sufficient versus deficient participants (Group 4 versus Group 1)		
Sphingomyelins	1.98 × 10^−36^	<0.001
Phosphatidylcholine (PC)	1.09 × 10^−16^	<0.001
Phosphatidylethanolamine (PE)	3.80 × 10^−12^	<0.001
Lysophospholipid	9.51 × 10^−10^	<0.001
Diacylglycerol	1.80 × 10^−5^	<0.001
Ceramides	2.42 × 10^−4^	0.004

## Data Availability

Restrictions apply to the availability of these data. Data was obtained from Qatar Biobank (https://www.qatarbiobank.org.qa, accessed on 12 August 2022) under confidentiality agreement with Qatar University. Supplementary Data are freely available.

## Supplementary Materials

The following supporting information can be downloaded at: https://www.mdpi.com/article/10.3390/metabo12080771/s1, Figure S1: Volcano plots of metabolites enrichment among participants’ groups. Vitamin D sufficient and normolipidemic (Group 1, n = 64); Vitamin D sufficient and Dyslipidemia (Group 2, n = 26); Vitamin D deficient and Dyslipidemia (Group 3, n = 88); Vitamin D deficient and normolipidemic (Group 4, n = 99); Table S1: Metabolites with significantly different levels in vitamin D sufficient groups (Group 2 versus Group 1) (Dyslipidemic versus normolipidemic participants); Table S2: Metabolites with significantly different levels in vitamin D deficient groups (Group 4 versus Group 3) (Normolipidemic versus dyslipidemic participants); Table S3: Metabolites with significantly different levels in normolipidemics groups (Group 4 versus Group 1) vitamin D deficient versus sufficient participants; Table S4: Metabolites with significantly different levels in dyslipidemics groups (group 3 versus group 2) vitamin D deficient versus sufficient participants; Appendix S1: The variable influence on projection (VIP) list indicating top metabolites that differentiate the four groups.
