# Supplementary material for: Metabolomics Profiling of Vitamin D Status in Relation to Dyslipidemia
Source: Metabolites. 2022 Aug 22;12(8):771. doi: 10.3390/metabo12080771 (PMC9416284; doi:10.3390/metabo12080771)
Supplement: Supplementary file 1 [file metabolites-12-00771-s001.zip › metabolites-1834510-supplementary.pdf]

Supplementary Materials

Metabolomics profiling of vitamin D status in relation to dyslipidemia

Hanaa Mousa[1], Mohamed A. Elrayess[2], Ilhame Diboun[3], Simon K. Jackson[4], Susu M. Zughaier[1]*

* Email correspondence: Susu M. Zughaier, szughaier@qu.edu.qa

Supplementary Figure S1

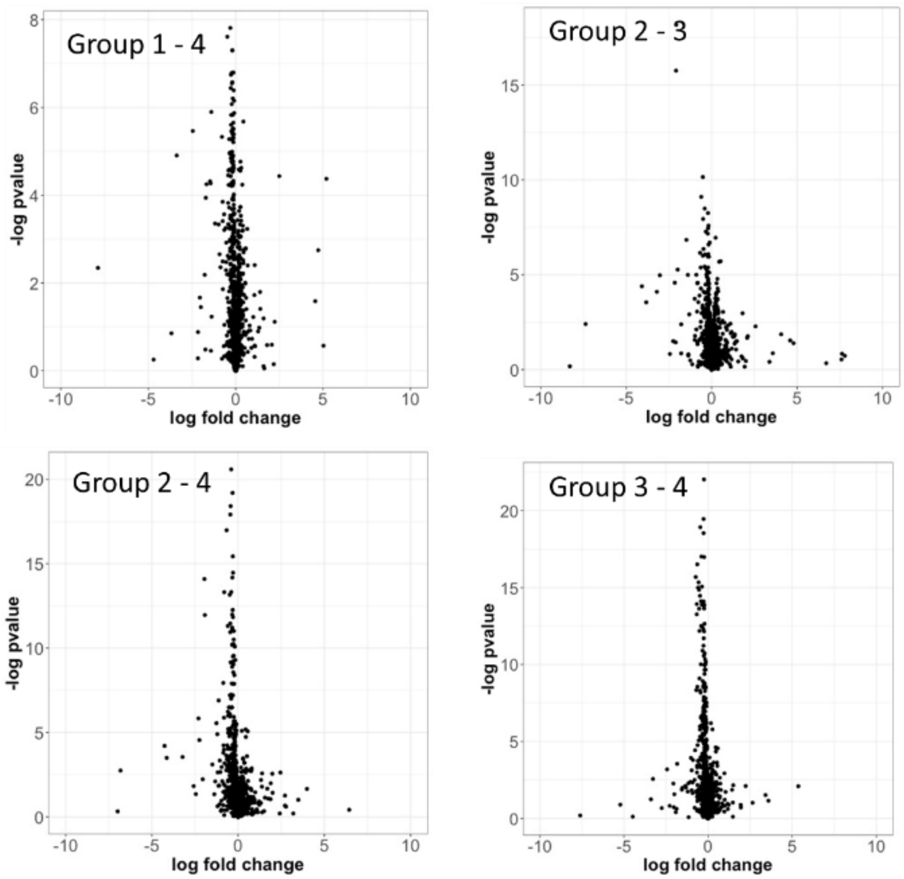

Figure S1: Volcano plots of metabolites enrichment among participants' groups. Vitamin D sufficient and normolipidemic (Group 1, n=64); Vitamin D sufficient and Dyslipidemia (Group 2, n=26); Vitamin D deficient and Dyslipidemia (Group 3, n=88); Vitamin D deficient and normolipidemic (Group 4, n=99).

Supplementary Table S1

Metabolites with significantly different levels in ***vitamin D sufficient groups*** (Group 2 versus Group 1) (Dyslipidemic versus *n*ormolipidemic participants)					
Metabolite Names	Sub-Pathway	Estimate	Std.Error	P.value	FDR
sphingomyelin (d18:2/23:0, d18:1/23:1, d17:1/24:1)*	Sphingomyelins	0.27	0.06	$1.34E^{-05}$	*<0.001*
1-palmitoyl-2-docosahexaenoyl-GPE (16:0/22:6)*	Phosphatidylethanolamine (PE)	0.67	0.16	$2.71E^{-05}$	*<0.001*
Cholesterol	Sterol	0.22	0.05	$3.17E^{-05}$	*<0.001*
sphingomyelin (d18:2/21:0, d16:2/23:0)*	Sphingomyelins	0.29	0.07	$3.56E^{-05}$	*<0.001*
sphingomyelin (d18:1/24:1, d18:2/24:0)*	Sphingomyelins	0.22	0.05	$6.71E^{-05}$	*<0.001*
sphingomyelin (d18:1/21:0, d17:1/22:0, d16:1/23:0)*	Sphingomyelins	0.30	0.07	$8.17E^{-05}$	0.01
sphingomyelin (d18:1/22:1, d18:2/22:0, d16:1/24:1)*	Sphingomyelins	0.20	0.06	$2.91E^{-04}$	0.02
sphingomyelin (d18:2/16:0, d18:1/16:1)*	Sphingomyelins	0.19	0.05	$3.82E^{-04}$	0.02
sphingomyelin (d18:1/24:1, d18:2/24:0)*	Sphingomyelins	0.23	0.07	$5.90E^{-04}$	0.03
sphingomyelin (d18:2/23:1)*	Sphingomyelins	0.26	0.07	$7.16E^{-04}$	0.03
sphingomyelin (d18:2/24:1, d18:1/24:2)*	Sphingomyelins	0.20	0.06	$7.18E^{-04}$	0.03
1-oleoyl-2-docosahexaenoyl-GPC (18:1/22:6)*	Phosphatidylcholine (PC)	0.36	0.11	$1.31E^{-03}$	0.04
sphingomyelin (d17:1/16:0, d18:1/15:0, d16:1/17:0)*	Sphingomyelins	0.21	0.06	$1.31E^{-03}$	0.04
sphingomyelin (d18:1/20:0, d16:1/22:0)*	Sphingomyelins	0.21	0.06	$1.55E^{-03}$	0.05
hydroxy-CMPF*	Fatty Acid, Dicarboxylate	1.17	0.37	$1.55E^{-03}$	0.05
3-carboxy-4-methyl-5-propyl-2-furanpropanoate (CMPF)	Fatty Acid, Dicarboxylate	1.27	0.40	$1.57E^{-03}$	0.05
sphingomyelin (d18:1/20:1, d18:2/20:0)*	Sphingomyelins	0.18	0.06	$1.59E^{-03}$	0.05
sphingomyelin (d18:2/14:0, d18:1/14:1)*	Sphingomyelins	0.24	0.07	$1.60E^{-03}$	0.05
behenoyl sphingomyelin (d18:1/22:0)*	Sphingomyelins	0.20	0.06	$1.80E^{-03}$	0.05
palmitoyl dihydrosphingomyelin (d18:0/16:0)*	Dihydrosphingomyelins	0.21	0.07	$1.81E^{-03}$	0.05

Supplementary Table S2

Metabolites with significantly different levels in ***vitamin D deficient groups*** (Group 4 versus Group 3) (Normolipidemic versus dyslipidemic participants)					
Metabolite Names	Sub-Pathway	Estimate	Std.Error	P.value	FDR
Cholesterol	Sterol	-0.24	0.04	$2.70E^{-10}$	*<0.001*
N-palmitoyl-sphinganine (d18:0/16:0)	Ceramides	-0.27	0.04	$3.54E^{-09}$	*<0.001*
1-palmitoyl-2-dihomo-linolenoyl-GPC (16:0/20:3n3 or 6)*	Phosphatidylcholine (PC)	-0.46	0.08	$6.03E^{-09}$	*<0.001*
1-stearoyl-GPC (18:0)	Lysophospholipid	-0.27	0.05	$8.86E^{-09}$	*<0.001*
1-stearoyl-GPE (18:0)	Lysophospholipid	-0.40	0.07	$4.08E^{-08}$	*<0.001*
1-palmitoyl-GPC (16:0)	Lysophospholipid	-0.24	0.04	$4.21E^{-08}$	*<0.001*
1-myristoyl-2-arachidonoyl-GPC (14:0/20:4)*	Phosphatidylcholine (PC)	-0.65	0.12	$6.73E^{-08}$	*<0.001*
1-stearoyl-2-oleoyl-GPE (18:0/18:1)	Phosphatidylethanolamine (PE)	-0.74	0.14	$1.54E^{-07}$	*<0.001*
oleoyl-linoleoyl-glycerol (18:1/18:2) [2]	Diacylglycerol	-0.57	0.11	$2.18E^{-07}$	*<0.001*
1-linoleoyl-2-arachidonoyl-GPC (18:2/20:4n6)*	Phosphatidylcholine (PC)	-0.37	0.07	$2.87E^{-07}$	*<0.001*
1-palmitoyl-2-linoleoyl-GPE (16:0/18:2)	Phosphatidylethanolamine (PE)	-0.60	0.11	$3.06E^{-07}$	*<0.001*
1-linolenoyl-GPC (18:3)*	Lysophospholipid	-0.54	0.10	$3.53E^{-07}$	*<0.001*
1-stearoyl-2-linoleoyl-GPE (18:0/18:2)*	Phosphatidylethanolamine (PE)	-0.48	0.09	$5.21E^{-07}$	*<0.001*
1-stearoyl-2-oleoyl-GPC (18:0/18:1)	Phosphatidylcholine (PC)	-0.43	0.08	$7.40E^{-07}$	*<0.001*
sphingomyelin (d18:1/21:0, d17:1/22:0, d16:1/23:0)*	Sphingomyelins	-0.27	0.05	$7.49E^{-07}$	*<0.001*
1-myristoyl-2-palmitoyl-GPC (14:0/16:0)	Phosphatidylcholine (PC)	-0.68	0.13	$8.95E^{-07}$	*<0.001*
1-palmitoleoyl-GPC (16:1)*	Lysophospholipid	-0.37	0.07	$9.32E^{-07}$	*<0.001*
behenoyl sphingomyelin (d18:1/22:0)*	Sphingomyelins	-0.23	0.05	$9.32E^{-07}$	*<0.001*
retinol (Vitamin A)	Vitamin A Metabolism	-0.26	0.05	$9.77E^{-07}$	*<0.001*
oleoyl-linoleoyl-glycerol (18:1/18:2) [1]	Diacylglycerol	-0.54	0.11	$1.20E^{-06}$	*<0.001*
1-oleoyl-GPC (18:1)	Lysophospholipid	-0.24	0.05	$1.19E^{-06}$	*<0.001*

1-palmitoyl-2-oleoyl-GPE (16:0/18:1)	Phosphatidylethanolamine (PE)	-0.68	0.14	$1.74E^{-06}$	*<0.001*
alpha-tocopherol	Tocopherol Metabolism	-0.21	0.04	$3.20E^{-06}$	*<0.001*
1-linoleoyl-GPE (18:2)*	Lysophospholipid	-0.41	0.09	$3.68E^{-06}$	*<0.001*
1-palmitoyl-GPE (16:0)	Lysophospholipid	-0.36	0.08	$3.87E^{-06}$	*<0.001*
1-arachidonoyl-GPE (20:4n6)*	Lysophospholipid	-0.29	0.06	$4.49E^{-06}$	*<0.001*
1-palmitoyl-2-palmitoleoyl-GPC (16:0/16:1)*	Phosphatidylcholine (PC)	-0.52	0.11	$5.11E^{-06}$	*<0.001*
sphingomyelin (d18:1/14:0, d16:1/16:0)*	Sphingomyelins	-0.24	0.05	$5.27E^{-06}$	*<0.001*
1-palmitoyl-2-oleoyl-GPC (16:0/18:1)	Phosphatidylcholine (PC)	-0.26	0.06	$8.22E^{-06}$	*<0.001*
1-stearoyl-2-arachidonoyl-GPC (18:0/20:4)	Phosphatidylcholine (PC)	-0.28	0.06	$1.33E^{-05}$	*<0.001*
1-stearoyl-2-arachidonoyl-GPE (18:0/20:4)	Phosphatidylethanolamine (PE)	-0.33	0.08	$1.84E^{-05}$	*<0.001*
sphingomyelin (d18:1/20:0, d16:1/22:0)*	Sphingomyelins	-0.20	0.05	$2.19E^{-05}$	*<0.001*
1-palmitoyl-2-arachidonoyl-GPC (16:0/20:4n6)	Phosphatidylcholine (PC)	-0.25	0.06	$2.56E^{-05}$	*<0.001*
sphingomyelin (d18:2/23:0, d18:1/23:1, d17:1/24:1)*	Sphingomyelins	-0.19	0.04	$3.20E^{-05}$	*<0.001*
Tryptophan	Tryptophan Metabolism	-0.15	0.04	$3.77E^{-05}$	*<0.001*
Glycerophosphoethanolamine	Phospholipid Metabolism	-0.17	0.04	$4.42E^{-05}$	*<0.001*
1-palmitoyl-2-arachidonoyl-GPE (16:0/20:4)*	Phosphatidylethanolamine (PE)	-0.43	0.10	$4.48E^{-05}$	*<0.001*
sphingomyelin (d18:2/14:0, d18:1/14:1)*	Sphingomyelins	-0.22	0.05	$4.60E^{-05}$	*<0.001*
1-linoleoyl-GPC (18:2)	Lysophospholipid	-0.20	0.05	$4.76E^{-05}$	*<0.001*
sphingomyelin (d17:1/16:0, d18:1/15:0, d16:1/17:0)*	Sphingomyelins	-0.19	0.05	$6.19E^{-05}$	*<0.001*
sphingomyelin (d18:1/22:1, d18:2/22:0, d16:1/24:1)*	Sphingomyelins	-0.16	0.04	$6.38E^{-05}$	*<0.001*
sphingomyelin (d18:2/21:0, d16:2/23:0)*	Sphingomyelins	-0.21	0.05	$6.54E^{-05}$	*<0.001*
1-arachidonoyl-GPC (20:4n6)*	Lysophospholipid	-0.21	0.05	$8.38E^{-05}$	0.01
linoleoyl-linoleoyl-glycerol (18:2/18:2) [1]*	Diacylglycerol	-0.52	0.13	$1.12E^{-04}$	0.01
glycosyl ceramide (d18:1/20:0, d16:1/22:0)*	Hexosylceramides (HCER)	-0.32	0.08	$1.33E^{-04}$	0.01

3-methyl-2-oxovalerate	Leucine, Isoleucine and Valine Metabolism	-0.21	0.05	$1.53E^{-04}$	0.01
sphingomyelin (d18:1/19:0, d19:1/18:0)*	Sphingomyelins	-0.21	0.06	$1.61E^{-04}$	0.01
4-methyl-2-oxopentanoate	Leucine, Isoleucine and Valine Metabolism	-0.21	0.06	$1.72E^{-04}$	0.01
glycosyl-N-stearoyl-sphingosine (d18:1/18:0)	Hexosylceramides (HCER)	-0.30	0.08	$1.86E^{-04}$	0.01
1-palmitoleoylglycerol (16:1)*	Monoacylglycerol	-0.64	0.17	$1.93E^{-04}$	0.01
sphingomyelin (d17:1/14:0, d16:1/15:0)*	Sphingomyelins	-0.27	0.07	$2.03E^{-04}$	0.01
sphingomyelin (d18:2/16:0, d18:1/16:1)*	Sphingomyelins	-0.15	0.04	$2.07E^{-04}$	0.01
1-linoleoylglycerol (18:2)	Monoacylglycerol	-0.70	0.19	$2.40E^{-04}$	0.01
1-stearoyl-GPE (18:0)	Lysophospholipid	-0.48	0.13	$2.48E^{-04}$	0.01
1,2-dipalmitoyl-GPC (16:0/16:0)	Phosphatidylcholine (PC)	-0.19	0.05	$2.55E^{-04}$	0.01
1-palmitoyl-GPI (16:0)	Lysophospholipid	-0.44	0.12	$2.83E^{-04}$	0.01
1-stearoyl-2-arachidonoyl-GPI (18:0/20:4)	Phosphatidylinositol (PI)	-0.16	0.04	$4.06E^{-04}$	0.02
sphingomyelin (d18:1/17:0, d17:1/18:0, d19:1/16:0)	Sphingomyelins	-0.18	0.05	$4.27E^{-04}$	0.02
palmitoylcarnitine (C16)	Fatty Acid Metabolism (Acyl Carnitine, Long Chain Saturated)	-0.26	0.07	$4.56E^{-04}$	0.02
1-stearoyl-2-docosahexaenoyl-GPC (18:0/22:6)	Phosphatidylcholine (PC)	-0.25	0.07	$4.80E^{-04}$	0.02
1-stearoyl-2-linoleoyl-GPC (18:0/18:2)*	Phosphatidylcholine (PC)	-0.14	0.04	$5.33E^{-04}$	0.02
glycerophosphorylcholine (GPC)	Phospholipid Metabolism	-0.09	0.03	$5.46E^{-04}$	0.02
Indoleacetate	Tryptophan Metabolism	-0.19	0.06	$6.00E^{-04}$	0.03
myristoyl dihydrosphingomyelin (d18:0/14:0)*	Dihydrosphingomyelins	-0.22	0.06	$6.38E^{-04}$	0.03
Leucine	Leucine, Isoleucine and Valine Metabolism	-0.10	0.03	$6.84E^{-04}$	0.03
N-stearoyl-sphingosine (d18:1/18:0)*	Ceramides	-0.27	0.08	$7.25E^{-04}$	0.03
1-palmitoyl-2-arachidonoyl-GPI (16:0/20:4)*	Phosphatidylinositol (PI)	-0.28	0.08	$7.48E^{-04}$	0.03
1-(1-enyl-stearoyl)-2-linoleoyl-GPE (P-18:0/18:2)*	Plasmalogen	-0.27	0.08	$8.48E^{-04}$	0.03

1-palmitoyl-2-docosahexaenoyl-GPC (16:0/22:6)	Phosphatidylcholine (PC)	-0.23	0.07	$8.98E^{-04}$	0.03
sphingomyelin (d18:2/18:1)*	Sphingomyelins	-0.17	0.05	$9.54E^{-04}$	0.04
1-arachidonoyl-GPI (20:4)*	Lysophospholipid	-0.29	0.09	$9.82E^{-04}$	0.04
isovalerylcarnitine (C5)	Leucine, Isoleucine and Valine Metabolism	-0.33	0.10	$1.20E^{-03}$	0.04
1-palmitoyl-2-linoleoyl-GPI (16:0/18:2)	Phosphatidylinositol (PI)	-0.23	0.07	$1.22E^{-03}$	0.04
N-palmitoyl-sphingadienine (d18:2/16:0)*	Dihydroceramides	-0.41	0.13	$1.33E^{-03}$	0.04
glycosyl-N-palmitoyl-sphingosine (d18:1/16:0)	Hexosylceramides (HCER)	-0.17	0.05	$1.32E^{-03}$	0.04
dihomo-linolenoyl-choline	Fatty Acid Metabolism (Acyl Choline)	-0.43	0.13	$1.46E^{-03}$	0.05
xanthure.te	Tryptophan Metabolism	-0.34	0.11	$1.55E^{-03}$	0.05
N-acetylneurami.te	Aminosugar Metabolism	-0.20	0.06	$1.55E^{-03}$	0.05
1-palmitoyl-2-linoleoyl-GPC (16:0/18:2)	Phosphatidylcholine (PC)	-0.13	0.04	$1.60E^{-03}$	0.05
1-oleoyl-2-docosahexaenoyl-GPC (18:1/22:6)*	Phosphatidylcholine (PC)	-0.26	0.08	$1.66E^{-03}$	0.05
1,2-dilinoleoyl-GPC (18:2/18:2)	Phosphatidylcholine (PC)	-0.32	0.10	$1.79E^{-03}$	0.05
sphingomyelin (d17:2/16:0, d18:2/15:0)*	Sphingomyelins	-0.18	0.06	$1.83E^{-03}$	0.05

Supplementary Table S3

Metabolites with significantly different levels in ***normolipidemics groups*** (Group 4 versus Group 1) vitamin D deficient versus sufficient participants					
Metabolite Names	Sub-Pathway	Estimate	Std.Error	P.value	FDR
1-oleoyl-2-docosahexaenoyl-GPC (18:1/22:6)*	Phosphatidylcholine (PC)	-0.31	0.09	$4.04E^{-04}$	0.02
Ergothioneine	Food Component/Plant	-0.48	0.13	$4.94E^{-04}$	0.02
Urea	Urea cycle; Arginine and Proline Metabolism	-0.19	0.05	6.73E-04	0.03
4-methyl-2-oxopentanoate	Leucine, Isoleucine and Valine Metabolism	-0.20	0.06	$6.76E^{-04}$	0.03
Valine	Leucine, Isoleucine and Valine Metabolism	-0.13	0.04	$1.12E^{-03}$	0.04
arabo.te/xylo.te	Pentose Metabolism	-0.23	0.07	1.13E-03	0.04
1-(1-enyl-oleoyl)-GPE (P-18:1)*	Lysoplasmalogen	-0.27	0.08	$1.18E^{-03}$	0.04
Allantoin	Purine Metabolism, (Hypo)Xanthine/Inosine containing	-0.19	0.06	$1.39E^{-03}$	0.05
methionine sulfone	Methionine, Cysteine, SAM and Taurine Metabolism	-0.22	0.07	$1.43E^{-03}$	0.05
1-(1-enyl-stearoyl)-GPE (P-18:0)*	Lysoplasmalogen	-0.28	0.09	$1.59E^{-03}$	0.05
Tryptophan	Tryptophan Metabolism	-0.12	0.04	$1.68E^{-03}$	0.05

Supplementary Table S4

Metabolites with significantly different levels in ***dyslipidemics groups*** (group 3 versus group 2) vitamin D deficient versus sufficient participants					
Metabolite Names	Sub-Pathway	Estimate	Std.Error	P.value	FDR
hydroxy-CMPF*	Fatty Acid, Dicarboxylate	-2.08	-5.87	$1.28E^{-08}$	*<0.001*
3-carboxy-4-methyl-5-propyl-2-furanpropanoate (CMPF)	Fatty Acid, Dicarboxylate	-2.09	-5.41	$1.44E^{-07}$	*<0.001*
docosahexaenoate (DHA; 22:6n3)	Long Chain Polyunsaturated Fatty Acid (n3 and n6)	-0.52	-4.18	$3.90E^{-05}$	*<0.001*
S-methylcysteine sulfoxide	Methionine, Cysteine, SAM and Taurine Metabolism	-0.62	-3.92	$1.11E^{-04}$	0.01
1-oleoyl-2-docosahexaenoyl-GPC (18:1/22:6)*	Phosphatidylcholine (PC)	-0.41	-3.76	$2.05E^{-04}$	0.01
sphingomyelin (d18:2/23:0, d18:1/23:1, d17:1/24:1)*	Sphingomyelins	-0.22	-3.70	$2.61E^{-04}$	0.01
perfluorooctanoate (PFOA)	Chemical	-0.51	-3.62	$3.56E^{-04}$	0.02
sphingomyelin (d18:1/24:1, d18:2/24:0)*	Sphingomyelins	-0.19	-3.52	$5.05E^{-04}$	0.02
hydroxypalmitoyl sphingomyelin (d18:1/16:0(OH))**	Sphingomyelins	-0.21	-3.48	$5.81E^{-04}$	0.03
tartro.te (hydroxymalo.te)	Food Component/Plant	-0.34	-3.43	$6.93E^{-04}$	0.03
sphingomyelin (d18:2/21:0, d16:2/23:0)*	Sphingomyelins	-0.23	-3.40	$7.73E^{-04}$	0.03
2-hydroxyglutarate	Fatty Acid, Dicarboxylate	0.23	3.34	$9.57E^{-04}$	0.04
Lysine	Lysine Metabolism	-0.13	-3.27	$1.23E^{-03}$	0.04
sphingomyelin (d18:2/24:1, d18:1/24:2)*	Sphingomyelins	-0.18	-3.23	$1.38E^{-03}$	0.05
1-palmitoyl-2-docosahexaenoyl-GPE (16:0/22:6)*	Phosphatidylethanolamine (PE)	-0.48	-3.16	$1.73E^{-03}$	0.05

Appendix S1: The variable influence on projection (VIP) list indicating top metabolites that differentiate the four groups

**Primary ID**	**M1.VIP[3+1+0]**	**M1.VIP[3]cvSE * 2.44693**
cholesterol	2.36969	0.834622
topiramate(-0.209857)	2.36638	5.31995
metoprolol(0.366655)	2.36638	5.31995
Suciprofloxacin(0.0701788)	2.36638	5.31995
saccharin(0.316561)	2.36638	5.31995
bradykinin(0.297286)	2.36638	5.31995
bradykinin..hydroxy.pro.3.(0.327431)	2.36638	5.31995
bradykinin..des.arg.9.(0)	2.36638	5.31995
pioglitazone(0)	2.36638	5.31995
Fibrinopeptide.A..phosphono.ser.3..(0.125222)	2.36638	5.31995
HXGXA.(0.126016)	2.36638	5.31995
lidocaine(-0.151288)	2.36638	5.31995
carbamazepine(0.326278)	2.36638	5.31995
Nimesulide(0.538888)	2.36638	5.31995
nornicotine(0.808037)	2.36638	5.31995
carbamazepine.glucuronide.(0.411977)	2.36638	5.31995
oxypurinol(-0.0997098)	2.36638	5.31995
pseudoephedrine(0.0636319)	2.36638	5.31995
quetiapine(0.530628)	2.36638	5.31995
doxycycline(0.291325)	2.36638	5.31995
leucylglycine(0.10084)	2.36638	5.31995
mirtazapine(-0.691948)	2.36638	5.31995
verapamil(0.0993022)	2.36638	5.31995
furosemide(0.513362)	2.36638	5.31995
o.hydroxyatorvastatin(-0.459816)	2.36638	5.31995

olmesartan(0.173281)	2.36638	5.31995
daidzein.sulfate..2.(-0.101258)	2.36638	5.31995
alpha.hydroxymetoprolol(0.0978524)	2.36638	5.31995
rivaroxaban(1.3156)	2.36638	5.31995
tadalafil(0.157602)	2.36638	5.31995
cetirizine(0.485692)	2.36638	5.31995
mycophenolic.acid.glucuronide(-1.17151)	2.36638	5.31995
sulfamethoxazole(0.036814)	2.36638	5.31995
dexlansoprazole(0.859974)	2.36638	5.31995
metronidazole(0.154179)	2.36638	5.31995
moxifloxacin(0.432496)	2.36638	5.31995
fluco.zole(0.0695261)	2.36638	5.31995
THC.carboxylic.acid.glucuronide(0.698234)	2.36638	5.31995
resveratrol.disulfate..1..(0.127601)	2.36638	5.31995
montelukast(0.301289)	2.36638	5.31995
tamoxifen(-0.885761)	2.36638	5.31995
Urolithin.B.glucuronide(-0.0322133)	2.36638	5.31995
Gliclazide(1.39632)	2.36638	5.31995
X...12407(-0.316905)	2.36638	5.31995
X...16649(0.141065)	2.36638	5.31995
X...21315(-0.458233)	2.36638	5.31995
X...24576(0.142974)	2.36638	5.31995
X...25503(-0.0144032)	2.36638	5.31995
X...25523(-0.355961)	2.36638	5.31995
X1.stearoyl.2.oleoyl.GPE..18.0.18.1.	2.34805	0.694351
X1.stearoyl.GPC..18.0.	2.25932	0.330957
sphingomyelin..d18.1.17.0..d17.1.18.0..d19.1.16.0.	2.25362	0.943465
sphingomyelin..d18.1.19.0..d19.1.18.0..	2.25154	1.05206

sphingomyelin..d18.1.24.1..d18.2.24.0..	2.21323	0.679924
X1.stearoyl.2.oleoyl.GPC..18.0.18.1.	2.20914	0.466183
sphingomyelin..d18.2.23.0..d18.1.23.1..d17.1.24.1..	2.19259	1.129
X1.palmitoyl.2.dihomo.linolenoyl.GPC..16.0.20.3n3.or.6..	2.18576	0.390977
oleoyl.linoleoyl.glycerol..18.1.18.2...2.	2.18444	0.403899
X1.myristoyl.2.arachidonoyl.GPC..14.0.20.4..	2.18213	0.232895
sphingomyelin..d18.2.21.0..d16.2.23.0..	2.17481	0.949588
sphingomyelin..d17.2.16.0..d18.2.15.0..	2.1747	0.621318
X1.palmitoyl.2.docosahexaenoyl.GPE..16.0.22.6..	2.17153	0.690187
sphingomyelin..d18.1.20.0..d16.1.22.0..	2.16443	0.948554
hydroxypalmitoyl.sphingomyelin..d18.1.16.0.OH....	2.15139	0.653774
X1.stearoyl.2.arachidonoyl.GPC..18.0.20.4.	2.14214	0.330713
behenoyl.sphingomyelin..d18.1.22.0..	2.11965	0.823317
retinol..Vitamin.A.	2.1121	0.413701
X1.palmitoyl.2.oleoyl.GPE..16.0.18.1.	2.08933	0.249534
picolinoylglycine	2.08832	0.401619
X1.palmitoyl.GPC..16.0.	2.08712	0.396868
X1.palmitoyl.2.palmitoleoyl.GPC..16.0.16.1..	2.08207	0.577945
X1.palmitoyl.2.arachidonoyl.GPC..16.0.20.4n6.	2.07972	0.229758
N.palmitoyl.sphingosine..d18.1.16.0.	2.07575	0.734881
sphingomyelin..d18.1.14.0..d16.1.16.0..	2.07486	0.769818
X1.oleoyl.2.docosahexaenoyl.GPC..18.1.22.6..	2.06912	0.703712
X1.palmitoyl.2.oleoyl.GPC..16.0.18.1.	2.05812	0.47857
X1.myristoyl.2.palmitoyl.GPC..14.0.16.0.	2.04785	0.424867
sphingomyelin..d17.1.14.0..d16.1.15.0..	2.04485	0.566294
X1.palmitoyl.GPE..16.0.	2.03499	0.400479
X1.arachidonoyl.GPE..20.4n6..	2.03397	0.268434

sphingomyelin..d17.1.16.0..d18.1.15.0..d16.1.17.0..	2.03386	1.15378
X1.palmitoleoylglycerol..16.1..	2.01192	0.551641
X1.palmitoyl.2.arachidonoyl.GPE..16.0.20.4..	2.00944	0.28702
X2.stearoyl.GPE..18.0..	2.00663	0.702131
tricosanoyl.sphingomyelin..d18.1.23.0..	2.00171	0.699729
X3.carboxy.4.methyl.5.propyl.2.furanpropanoate..CMPF.	1.99954	0.89435
docosahexaenoate..DHA..22.6n3.	1.99939	0.450131
X1.palmitoleoyl.GPC..16.1..	1.99611	0.386379
gamma.glutamylvaline	1.98876	0.540959
X2.3.dihydroxy.5.methylthio.4.pentenoate..DMTPA..	1.98266	0.467238
glutamate	1.98161	0.763551
oleoyl.linoleoyl.glycerol..18.1.18.2...1.	1.98099	0.619825
gamma.glutamylisoleucine.	1.97329	0.505567
sphingomyelin..d18.1.22.1..d18.2.22.0..d16.1.24.1..	1.95528	1.13785
sphingomyelin..d18.2.24.1..d18.1.24.2..	1.95237	0.805336
X1.linolenoyl.GPC..18.3..	1.94059	0.542771
isoleucine	1.92013	0.443313
leucine	1.91238	0.479729
palmitoyl.sphingomyelin..d18.1.16.0.	1.91198	0.642666
stearoyl.sphingomyelin..d18.1.18.0.	1.90657	0.711397
sphingomyelin..d18.2.23.1..	1.9059	0.862992
topiramate(NA)	1.90062	1.87973
ciprofloxacin(NA)	1.90062	1.87973
carbamazepine(NA)	1.90062	1.87973
carbamazepine.glucuronide.(NA)	1.90062	1.87973
quetiapine(NA)	1.90062	1.87973
mirtazapine(NA)	1.90062	1.87973

verapamil(NA)	1.90062	1.87973
olmesartan(NA)	1.90062	1.87973
rivaroxaban(NA)	1.90062	1.87973
moxifloxacin(NA)	1.90062	1.87973
fluco.zole(NA)	1.90062	1.87973
tamoxifen(NA)	1.90062	1.87973
X1.linoleoylglycerol..18.2.	1.89866	0.673765
X1.stearoyl.2.linoleoyl.GPE..18.0.18.2..	1.89826	0.513955
X1.2.dipalmitoyl.GPC..16.0.16.0.	1.89354	0.342115
creatinine	1.88847	0.477747
gamma.glutamylleucine	1.88607	0.542556
eicosapentaenoate..EPA..20.5n3.	1.88362	0.64662
indolelactate	1.87905	0.633497
X1.stearoyl.2.arachidonoyl.GPE..18.0.20.4.	1.87793	0.327332
hydroxy.CMPF.	1.87781	0.976351
X1.linoleoyl.2.arachidonoyl.GPC..18.2.20.4n6..	1.86847	0.216547
X...14056	1.86635	0.671933
ergothioneine	1.86058	0.755894
myristoyl.dihydrosphingomyelin..d18.0.14.0..	1.85797	0.488927
isovalerylcarnitine..C5.	1.85412	0.557548
X1.carboxyethylphenylalanine	1.84358	0.555876
N.acetyltryptophan	1.83292	0.644565
X1.palmitoyl.2.arachidonoyl.GPI..16.0.20.4..	1.8236	0.673409
X1.palmitoyl.2.linoleoyl.GPE..16.0.18.2.	1.82163	0.350314
valine	1.82056	0.410469
N.acetylcarnosine	1.8036	0.667713
N.acetylphenylalanine	1.80295	0.679274
X...11381	1.8023	1.45512

urate	1.80142	0.717666
X1.arachidonoyl.GPC..20.4n6..	1.79774	0.30015
kynurenine	1.7903	0.40859
xanthure.te	1.78498	0.389665
alpha.tocopherol	1.77046	0.616832
N.palmitoyl.sphingadienine..d18.2.16.0..	1.76538	0.534657
doxycycline(NA)	1.76033	1.52999
sulfamethoxazole(NA)	1.76033	1.52999
tartro.te..hydroxymalo.te.	1.75923	0.749404
X2.oxoarginine.	1.75436	0.468637
X1.arachidonoyl.GPI..20.4..	1.74616	0.908589
o.hydroxyatorvastatin(NA)	1.74601	1.60324
X...11372	1.7447	0.868739
palmitoyl.dihydrosphingomyelin..d18.0.16.0..	1.74418	0.695616
tryptophan	1.74057	0.377358
X3..4.hydroxyphenyl.lactate	1.73895	0.678754
X5alpha.preg.n.3beta.20alpha.diol.disulfate	1.73156	0.537951
X1.ribosyl.imidazoleacetate.	1.73054	0.39182
N.stearoyl.sphingosine..d18.1.18.0..	1.72911	1.09875
X1.oleoyl.GPE..18.1.	1.72757	0.492827
sphingomyelin..d18.1.18.1..d18.2.18.0.	1.72373	0.935222
mycophenolic.acid.glucuronide(NA)	1.72246	1.86886
X1.arachidonylglycerol..20.4.	1.72214	0.596492
sphingomyelin..d18.0.20.0..d16.0.22.0..	1.71836	0.602218
oxalate..ethanedioate.	1.71314	0.762046
cerotoylcarnitine..C26..	1.71204	0.327408
X1.oleoyl.GPC..18.1.	1.70951	0.573724
aspartate	1.70655	0.645563

gamma.glutamylphenylalanine	1.70617	0.401629
X...12117	1.7008	0.652746
X...12407(NA)	1.69479	1.49337
X...25371	1.69143	0.532042
X2.methylbutyrylcarnitine..C5.	1.68855	0.394336
X1.palmitoyl.GPI..16.0.	1.6861	0.758535
N6.N6.N6.trimethyllysine	1.68561	0.353946
X2.aminoadipate	1.68478	0.747301
gamma.glutamylglutamate	1.68361	0.578132
sphingomyelin..d18.2.16.0..d18.1.16.1..	1.67504	1.37883
X...23636	1.675	0.36457
X1.linoleoyl.GPE..18.2..	1.67348	0.408356
hydroxy.N6.N6.N6.trimethyllysine.	1.67143	0.364349
glycerate	1.66905	0.507691
N.acetylisoleucine	1.66623	0.145291
X1.linolenoylglycerol..18.3.	1.65435	0.428828
cys.gly..oxidized	1.65427	0.396388
beta.cryptoxanthin	1.65354	1.1428
N.acetylleucine	1.65341	0.383118
X2.hydroxy.4..methylthio.butanoic.acid	1.65046	0.749371
N.acetylvaline	1.64899	0.295912
X...24337	1.64876	0.388895
sphingomyelin..d18.2.14.0..d18.1.14.1..	1.64695	0.69867
N1.methyladenosine	1.64003	0.637506
X3.methyl.2.oxovalerate	1.63922	0.83924
X...13553	1.63093	0.561989
X2.O.methylascorbic.acid	1.62789	0.80382
palmitoylcarnitine..C16.	1.62702	0.464147

X1.stearoyl.GPI..18.0.	1.6248	0.907513
sphingomyelin..d18.1.22.2..d18.2.22.1..d16.1.24.2..	1.62427	0.91522
X...13866	1.62141	0.920365
X...12100	1.61581	0.419364
sphingomyelin..d18.1.20.1..d18.2.20.0..	1.61414	1.27564
X4.hydroxyglutamate	1.61078	0.402713
X2.hydroxy.3.methylvalerate	1.60805	0.871152
phenyllactate..PLA.	1.60749	0.556237
hydroxyasparagine..	1.59656	0.694661
X5alpha.preg.n.3beta.20beta.diol.monosulfate..1.	1.59492	0.644417
proline	1.59129	0.476797
X...11880	1.58999	0.833707
X4.methyl.2.oxopentanoate	1.58933	0.776015
linoleoyl.linoleoyl.glycerol..18.2.18.2...1..	1.58595	0.191327
glycerophosphoethanolamine	1.58077	0.539975
X5alpha.preg.n.3beta.20alpha.diol.monosulfate..2.	1.57783	0.602048
tiglylcarnitine..C5.1.DC.	1.57341	0.654356
X1.dihomo.linolenylglycerol..20.3.	1.57119	0.372718
S.methylcysteine.sulfoxide	1.57102	1.00462
X3.carboxy.4.methyl.5.pentyl.2.furanpropio.te..3.CMPFP...	1.56741	1.14625
phenylalanine	1.56668	0.462731
X10.undecenoate..11.1n1.	1.56379	0.673739
histidine.betaine..hercynine..	1.56236	0.605237
X5alpha.androstan.3alpha.17beta.diol.disulfate	1.56215	0.512361
X17alpha.hydroxypregnenolone.3.sulfate	1.56082	0.462116
X7.methylguanine	1.55889	0.47284
cystathionine	1.55776	0.527065

propionylcarnitine..C3.	1.55689	0.466512
N.acetylglycine	1.55569	0.547298
X3.formylindole	1.55023	0.192424
pseudoephedrine(NA)	1.54928	0.626332
X...23593	1.54777	0.622125
S.adenosylhomocysteine..SAH.	1.54635	0.562772
alpha.hydroxyisovalerate	1.54389	0.741083
X...24951	1.54174	0.679852
X5alpha.preg.n.diol.disulfate	1.54154	0.617473
X2.palmitoyl.GPC..16.0..	1.54051	0.746586
arabo.te.xylo.te	1.53759	1.11985
X...21319	1.5317	0.906947
X4.hydroxyphenylacetylglutamine	1.53092	0.454556
argini.te.	1.5294	0.579401
X3.phosphoglycerate	1.5292	0.618358
X...13684	1.52761	0.654522
isobutyrylcarnitine..C4.	1.52761	0.346793
X5.methylthioadenosine..MTA.	1.52649	0.554774
palmitoyl.ethanolamide	1.52492	0.881462
ascorbic.acid.3.sulfate.	1.52114	0.647338
X1.3.dimethylurate	1.5207	0.379378
X...24588	1.52055	0.594804
montelukast(NA)	1.51775	2.01045
nisi.te..24.6n3.	1.5152	0.333801
carotene.diol..2.	1.51298	1.3979
X4.methoxyphenol.sulfate	1.50915	0.406824
Nimesulide(NA)	1.50425	2.04746
X3.hydroxy.2.ethylpropio.te	1.49955	0.658845

carotene.diol..1.	1.49954	1.31531
eicosanedioate..C20.DC.	1.49933	0.905991
sphingosine.1.phosphate	1.49806	0.695652
EDTA	1.49629	0.392238
gamma.glutamyltyrosine	1.49608	0.273261
alpha.ketoglutarate	1.49585	1.03456
pregnenediol.disulfate..C21H34O8S2..	1.49519	0.676365
dexlansoprazole(NA)	1.49467	1.91361
X3.methyl.2.oxobutyrate	1.49459	0.458975
N2.N2.dimethylguanosine	1.49246	1.10417
X1.stearoyl.2.arachidonoyl.GPI..18.0.20.4.	1.48885	0.809644
glycerol.3.phosphate	1.48859	0.714194
X...19438	1.48618	0.648457
preg.nediol.3.glucuronide	1.48446	0.706895
X1.palmitoyl.2.oleoyl.GPI..16.0.18.1..	1.48205	0.985238
urea	1.4819	0.715778
X...16935	1.48152	0.806944
ornithine	1.48141	0.697112
X...11308	1.4804	1.02549
X1.palmitoyl.2.linoleoyl.GPI..16.0.18.2.	1.47905	0.903546
sphingomyelin..d18.0.18.0..d19.0.17.0..	1.47795	0.785233
eicosenedioate..C20.1.DC..	1.47701	0.660435
hexadecenedioate..C16.1.DC..	1.47468	0.679252
dihomo.linole.te..20.3n3.or.n6.	1.47361	0.520058
succinylcarnitine..C4.DC.	1.4725	0.206076
pregnenolone.sulfate	1.46558	1.02971
gamma.glutamyl.alpha.lysine	1.46491	0.444693
N1.methylinosine	1.46454	0.642763

X...17340	1.46375	0.548228
X...23639	1.46336	0.716549
arabitol.xylitol	1.4631	0.851965
X...23739	1.46278	0.792064
X...10458	1.46269	0.430949
X1..1.enyl.stearoyl..GPE..P.18.0..	1.46267	0.875511
cysteinylglycine	1.46096	0.432083
erythro.te.	1.45991	1.2116
X1.carboxyethylvaline	1.45503	0.52508
glycochenodeoxycholate.glucuronide..1.	1.45489	0.520447
X...24306	1.45466	0.599904
isovalerate..i5.0.	1.45203	0.330133
perfluorooctanesulfo.te..PFOS.	1.45169	0.565699
alpha.hydroxyisocaproate	1.44897	0.934658
X1.methylhistidine	1.44815	0.647982
X21.hydroxypregnenolone.disulfate	1.43597	0.405892
X1..1.enyl.stearoyl..2.arachidonoyl.GPE..P.18.0.20.4..	1.43583	0.451993
N.acetyltyrosine	1.43555	0.592279
hydroxybupropion(NA)	1.43246	1.58771
sarcosine	1.4237	0.34737
X1.methylguanidine	1.42342	0.514395
guaiacol.sulfate	1.42244	0.414717
alanine	1.42191	0.319832
X4.guanidinobutanoate	1.42188	0.562873
N2.N5.diacetylornithine	1.41946	0.664675
pantothe.te	1.41909	0.801667
sphingomyelin..d18.2.24.2..	1.41768	1.02116
octadecenedioate..C18.1.DC.	1.41222	0.745204

X...21339	1.40698	0.868741
glucuro.te	1.40675	0.548724
X...24576(NA)	1.40312	1.27763
X1..1.enyl.palmitoyl..2.arachidonoyl.GPE..P.16.0.20.4..	1.4009	0.379755
X1.lignoceroyl.GPC..24.0.	1.39624	0.325832
stearoylcarnitine..C18.	1.39265	0.575675
catechol.sulfate	1.3883	0.26045
kynure.te	1.38678	0.391083
Glc.c.sulfate.conjugate.of.C21H34O2.steroid..	1.38533	0.408187
X3.4.dihydroxybutyrate	1.38495	0.715614
methylsuccinoylcarnitine	1.38471	0.271972
X...14939	1.38418	0.734151
X5.hydroxyindoleacetate	1.38114	0.552812
pyruvate	1.38032	0.80954
methyl.indole.3.acetate	1.37995	0.445368
X1.methylurate	1.3773	0.317645
THC.carboxylic.acid.glucuronide(NA)	1.37404	1.35208
X...24422	1.37363	1.1334
sphingomyelin..d18.2.18.1..	1.37161	1.10928
N6.methyladenosine	1.37032	0.54713
X5..galactosylhydroxy..L.lysine	1.36983	0.693406
homocitrulline	1.36924	0.377573
N.formylmethionine	1.36858	0.799088
trigonelline..N..methylnicoti.te.	1.36817	0.448795
sphingomyelin..d18.1.20.2..d18.2.20.1..d16.1.22.2..	1.36788	0.834793
gamma.glutamyltryptophan	1.36695	0.454998
X1.stearoyl.GPS..18.0..	1.3654	0.434827
gamma.glutamylhistidine	1.35821	0.478048

androstenediol..3beta.17beta..disulfate..1.	1.35779	0.453993
pregnenediol.sulfate..C21H34O5S..	1.35675	0.735488
threo.te	1.35446	0.453153
X1.linoleoyl.GPC..18.2.	1.35357	0.642691
X...24295	1.35059	0.562764
pioglitazone(NA)	1.35048	0.909927
X1..1.enyl.palmitoyl..GPE..P.16.0..	1.34995	0.801862
picoli.te	1.34841	0.232229
X2R.3R.dihydroxybutyrate	1.34634	0.502985
glycosyl.N.stearoyl.sphingosine..d18.1.18.0.	1.34596	0.453882
deoxycholate	1.34547	0.468338
X...15503	1.34266	0.269036
glycocholate	1.34129	0.3819
ascorbic.acid.2.sulfate	1.33956	0.273657
prolylglycine	1.33756	0.648905
hydroxybupropion(0)	1.33618	2.71736
X11beta.hydroxyandrosterone.glucuronide	1.33538	0.509354
palmitoyl.sphingosine.phosphoethanolamine..d18.1.16.0.	1.33189	1.03763
X5.methyluridine..ribothymidine.	1.33063	0.616118
deoxycarnitine	1.32978	0.686815
gulo.te.	1.3293	0.784614
glycochole.te.sulfate.	1.32795	0.567508
X...24546	1.32689	0.762966
X...25948	1.32671	0.464584
methyl.glucopyranoside..alpha...beta.	1.32459	0.401401
S.carboxyethylcysteine	1.32324	0.878134
N6.carbamoylthreonyladenosine	1.32291	0.667301

X4.allylphenol.sulfate	1.3182	0.266559
cysteine	1.31794	0.369887
X3.amino.2.piperidone	1.31738	0.529176
metabolonic.lactone.sulfate	1.31723	0.836544
X...25828	1.312	0.692281
docosahexaenoylcholine	1.31198	0.476756
X...24494	1.30992	0.592001
vanillylmandelate..VMA.	1.30958	1.16039
eugenol.sulfate	1.30896	0.413488
serotonin	1.30823	0.642752
pregnenetriol.sulfate.	1.30345	0.519566
glycochenodeoxycholate.3.sulfate	1.29921	0.514108
maltose	1.29678	0.456909
pseudouridine	1.2956	0.788865
N.palmitoyl.sphinganine..d18.0.16.0.	1.29267	0.201323
X5.6.dihydrouridine	1.29254	0.679205
X4.acetamidobutanoate	1.29228	0.755173
X...11315	1.28594	0.968347
X1.linoleoyl.GPI..18.2..	1.28523	0.902363
X...24970	1.28168	0.327752
formiminoglutamate	1.28101	0.386892
X...24953	1.27926	0.830322
X3.5.dichloro.2.6.dihydroxybenzoic.acid	1.27507	0.370274
cysteinylglycine.disulfide.	1.27488	0.450874
X.S..a.amino.omega.caprolactam	1.27256	0.386029
manno.te.	1.27037	0.754086
N.acetylalanine	1.26788	0.594361
X...21733	1.26731	0.496758

phosphoethanolamine	1.26626	0.609998
furosemide(NA)	1.26575	1.03346
adenosine.5..monophosphate..AMP.	1.26564	0.369244
X2.hydroxypalmitate	1.2645	0.87042
hydantoin.5.propio.te	1.26042	0.547997
androstenediol..3alpha..17alpha..monosulfate..3.	1.25984	0.569473
X...16087	1.25939	0.394238
branched.chain.14.0.dicarboxylic.acid..	1.2569	0.682871
methionine.sulfoxide	1.25646	0.867933
X...15666	1.2561	0.442864
X...25271	1.2554	0.488316
N.N.N.trimethyl.alanylproline.betaine..TMAP.	1.25467	0.547325
carotene.diol..3.	1.2545	0.681629
X...24544	1.25316	0.360999
X1.linoleoyl.GPG..18.2..	1.24983	0.57319
pregnenetriol.disulfate.	1.24818	0.428575
X...11787	1.2478	0.93401
N.acetylputrescine	1.24776	0.625699
choline	1.24775	0.498191
N.acetylkynurenine..2.	1.24352	0.466669
arachido.te..20.4n6.	1.24322	0.582696
homostachydrine.	1.24263	0.653606
X1..1.enyl.stearoyl..2.oleoyl.GPE..P.18.0.18.1.	1.24223	0.664782
isobutyrylglycine	1.23834	0.331091
X...12844	1.23602	0.538304
glucose	1.2354	0.35817
nicoti.mide	1.23522	0.566717
pentose.acid.	1.23514	0.602754

X3.methoxytyrosine	1.23491	0.910159
X1.oleoyl.GPI..18.1.	1.23389	0.531263
dopamine.3.O.sulfate	1.23381	0.615629
metronidazole(NA)	1.23377	0.917157
X...24456	1.23312	0.408924
X...17346	1.23309	0.40597
theophylline	1.22967	0.447031
X5alpha.androstan.3beta.17beta.diol.disulfate	1.22957	0.471922
glycerophosphorylcholine..GPC.	1.2292	0.795688
pyroglutamine.	1.2292	0.453513
pipecolate	1.22665	0.496133
X3..3.hydroxyphenyl.propio.te.sulfate	1.22528	0.274789
paraxanthine	1.22288	0.463254
citrulline	1.22116	0.477745
methionine.sulfone	1.22073	0.555412
X...17654	1.22069	0.812961
caffeine	1.21983	0.635654
X1..1.enyl.oleoyl..GPE..P.18.1..	1.21895	1.13816
X...25519	1.21709	0.627664
glycosyl.ceramide..d18.1.20.0..d16.1.22.0..	1.21701	0.538177
N.methylpipecolate	1.21654	0.35119
alpha.hydroxymetoprolol(NA)	1.21615	0.687313
androstenediol..3beta.17beta..disulfate..2.	1.21569	0.411469
X3.hydroxyphenylacetoylglutamine	1.21388	0.494381
dimethylarginine..SDMA...ADMA.	1.21261	0.596006
X1.7.dimethylurate	1.21118	0.666377
N6.acetyllysine	1.21089	0.821744
androstenediol..3alpha..17alpha..monosulfate..2.	1.20995	0.598015

X1.methylxanthine	1.2099	0.599299
X...17653	1.20888	1.07938
X3.indoleglyoxylic.acid	1.20824	0.495257
metoprolol(NA)	1.20681	1.76881
X...17325	1.20633	0.376406
X4.hydroxyphenylacetate	1.20515	0.497452
adenine	1.20495	0.327636
N.acetylneurami.te	1.20182	0.311579
X2.6.dihydroxybenzoic.acid	1.20068	0.506348
X...21736	1.20027	1.23208
X...18913	1.19972	0.459977
X...23782	1.19677	0.984512
glycochenodeoxycholate	1.19674	0.410631
X2.linoleoylglycerol..18.2.	1.19658	0.449587
N.acetylproline	1.19515	0.669252
X7.alpha.hydroxy.3.oxo.4.cholestenoate..7.Hoca.	1.19395	0.397334
xanthine	1.19328	1.04081
pro.hydroxy.pro	1.1922	0.584365
X...21258	1.19201	0.353078
X...17010	1.18986	0.238395
myo.inositol	1.18874	0.458481
lysine	1.18618	0.74205
vanillactate	1.18396	0.720683
docosapentaenoate..n3.DPA..22.5n3.	1.17844	0.610313
X1.2.dilinoleoyl.GPC..18.2.18.2.	1.17808	0.53771
X...24241	1.17709	0.654936
hyocholate	1.1742	0.464021
X...22162	1.17357	1.02228

X...12306	1.1729	0.395415
glycodeoxycholate.3.sulfate	1.17164	0.383427
X1.methyl.4.imidazoleacetate	1.16891	0.292093
X3.methylglutarylcarnitine..2.	1.16764	0.535239
hippurate	1.16507	0.317799
cortisone	1.16475	0.374737
X2.hydroxyglutarate	1.16335	0.643082
X...18887	1.16333	0.245844
hexanoylcarnitine..C6.	1.15939	0.515108
X4.ethylphenylsulfate	1.15929	0.586302
trans.4.hydroxyproline	1.15882	0.465847
X...12027	1.15701	0.321464
X...21441	1.15609	0.890581
cortolone.glucuronide..1.	1.15264	0.585678
X...21471	1.15195	0.893832
choline.phosphate	1.1516	0.335755
X2.hydroxystearate	1.14985	1.07529
X21.hydroxypregnenolone.monosulfate..1.	1.14921	0.420562
cetirizine(NA)	1.1491	1.31236
X2.hydroxyarachidate.	1.14884	0.427313
N.acetyl.2.aminooctanoate.	1.14832	0.859015
tridecenedioate..C13.1.DC..	1.14606	0.5132
X...18345	1.14485	0.406509
no.decanoate..19.0.	1.14388	0.586393
allantoin	1.14009	1.00081
hexadecanedioate..C16.DC.	1.1395	0.437027
X1.3.7.trimethylurate	1.13763	0.6174
gamma.glutamylmethionine	1.1345	0.416407

quinoli.te	1.13375	0.745627
X...16964	1.13327	0.592086
X1..1.enyl.palmitoyl..GPC..P.16.0..	1.13281	1.37226
N.N.N.trimethyl.5.aminovalerate	1.132	0.289618
X...12822	1.13166	0.45399
trimethylamine.N.oxide	1.13031	0.511323
X2.hydroxyoctanoate	1.13025	0.544043
X3.hydroxyisobutyrate	1.12907	0.470339
X...21740	1.12318	0.477843
erythritol	1.12003	0.620861
X4.allylcatechol.sulfate	1.11811	0.318185
branched.chain..straight.chain..or.cyclopropyl.12.1.fatty.acid.	1.11791	1.04927
serine	1.11778	0.74191
X...24949	1.1135	0.748651
X3..3.amino.3.carboxypropyl.uridine.	1.1129	0.479819
midazolam(NA)	1.11272	1.59694
oleoylcarnitine..C18.1.	1.11171	0.64372
N.acetyl.1.methylhistidine.	1.11149	0.587476
phenylpyruvate	1.1113	0.397472
X...12104	1.11051	0.442466
fluoxetine(-2.17156)	1.10946	2.78228
topiramate(0.0442083)	1.10946	2.78228
metoprolol(0.819295)	1.10946	2.78228
ciprofloxacin(-0.0336602)	1.10946	2.78228
saccharin(-0.0616627)	1.10946	2.78228
phenolphthalein.beta.D.glucuronide(-0.766578)	1.10946	2.78228
bradykinin..hydroxy.pro.3.(-0.519362)	1.10946	2.78228

hydroxybupropion(0.211557)	1.10946	2.78228
pioglitazone(-0.498732)	1.10946	2.78228
Fibrinopeptide.A..phosphono.ser.3..(0.422781)	1.10946	2.78228
HXGXA.(-0.576787)	1.10946	2.78228
nifedipine(0.403931)	1.10946	2.78228
nifedipine(NA)	1.10946	2.78228
lidocaine(0.0890177)	1.10946	2.78228
carbamazepine(0.517245)	1.10946	2.78228
Nimesulide(0.453048)	1.10946	2.78228
carbamazepine.glucuronide.(-0.705422)	1.10946	2.78228
oxypurinol(-0.593121)	1.10946	2.78228
pseudoephedrine(0.090937)	1.10946	2.78228
quetiapine(0)	1.10946	2.78228
doxycycline(-0.028914)	1.10946	2.78228
leucylglycine(-0.677077)	1.10946	2.78228
oseltamivir.carboxylate..Ro.64.0802.02.(0.678237)	1.10946	2.78228
oseltamivir.carboxylate..Ro.64.0802.02.(NA)	1.10946	2.78228
mirtazapine(-0.613782)	1.10946	2.78228
histidylalanine(0.547775)	1.10946	2.78228
norfluoxetine(0.501563)	1.10946	2.78228
verapamil(-0.549393)	1.10946	2.78228
hydroxypioglitazone..M.IV.(-0.373095)	1.10946	2.78228
furosemide(0.407197)	1.10946	2.78228
olmesartan(0.508984)	1.10946	2.78228
daidzein.sulfate..2.(-0.521381)	1.10946	2.78228
alpha.hydroxymetoprolol(-0.514332)	1.10946	2.78228
rivaroxaban(-0.0497156)	1.10946	2.78228
tadalafil(-0.615076)	1.10946	2.78228

cetirizine(-0.043534)	1.10946	2.78228
mycophenolic.acid.glucuronide(-0.36687)	1.10946	2.78228
sulfamethoxazole(-0.657587)	1.10946	2.78228
dexlansoprazole(-0.315533)	1.10946	2.78228
metronidazole(-0.467447)	1.10946	2.78228
moxifloxacin(0.0859942)	1.10946	2.78228
fluco.zole(-0.460133)	1.10946	2.78228
THC.carboxylic.acid.glucuronide(-0.587887)	1.10946	2.78228
X6.hydroxywarfarin(0.44244)	1.10946	2.78228
montelukast(0.310495)	1.10946	2.78228
midazolam(0)	1.10946	2.78228
tamoxifen(0.429247)	1.10946	2.78228
Urolithin.B.glucuronide(0.136016)	1.10946	2.78228
Gliclazide(1.43127)	1.10946	2.78228
X...12407(0.655082)	1.10946	2.78228
X...16649(-0.0232686)	1.10946	2.78228
X...21315(0.290578)	1.10946	2.78228
X...21842(-0.285418)	1.10946	2.78228
X...24576(0.479768)	1.10946	2.78228
X...25503(-0.0371828)	1.10946	2.78228
X...25523(0.0912109)	1.10946	2.78228
sulfate.	1.10936	0.44479
adenosine.3..5..cyclic.monophosphate..cAMP.	1.10892	0.377656
docosadioate..C22.DC.	1.10714	0.586305
margarate..17.0.	1.10282	0.919823
X1.5.anhydroglucitol..1.5.AG.	1.10246	0.399716
X3.indoxyl.sulfate	1.10037	0.304384
N1.Methyl.2.pyridone.5.carboxamide	1.0984	0.578406

deoxycholic.acid.glucuronide	1.09757	0.393138
indoleacetate	1.09755	0.791775
preg.nolone.allopreg.nolone.sulfate	1.09596	0.427877
Urolithin.B.glucuronide(NA)	1.09431	0.41614
X...11478	1.09418	0.892902
N.acetylarginine	1.09402	0.757645
HXGXA.(NA)	1.09326	0.701779
stearate..18.0.	1.09278	0.847773
caffeic.acid.sulfate	1.09077	0.813615
X...23587	1.088	0.917051
X...17146	1.08709	0.392895
X2..phthol.sulfate	1.0868	0.402715
X...11632	1.08612	0.434541
oleoyl.ethanolamide	1.08559	1.31901
homoarginine	1.08168	0.367006
X...17676	1.08042	0.380519
X...07765	1.07866	0.534101
stachydrine	1.07861	0.538876
X...18921	1.07851	1.167
erucate..22.1n9.	1.07772	0.781386
palmitate..16.0.	1.07704	0.996066
X1.linoleoyl.2.linolenoyl.GPC..18.2.18.3..	1.07307	0.598724
carnitine	1.07272	0.267359
leucylglycine(NA)	1.07166	0.640691
lactosyl.N.palmitoyl.sphingosine..d18.1.16.0.	1.07135	0.932486
p.hydroxybenzaldehyde	1.07127	0.646024
X3.hydroxystachydrine.	1.07001	0.662358
X5.acetylamino.6.amino.3.methyluracil	1.06987	0.638307

betonicine	1.06818	0.520023
N.acetylthreonine	1.06648	0.811193
X1.stearoyl.2.linoleoyl.GPC..18.0.18.2..	1.06518	0.278429
N.delta.acetylornithine	1.06418	0.330684
undecenoylcarnitine..C11.1.	1.05785	0.53339
X2.hydroxydecanoate	1.05755	0.263723
X3.hydroxypyridine.sulfate	1.05393	0.579843
laurylcarnitine..C12.	1.05307	0.631852
dodecadienoate..12.2..	1.05173	1.60148
X6.hydroxyindole.sulfate	1.05047	0.383459
octadecadienedioate..C18.2.DC..	1.04898	0.848092
X...16580	1.0486	0.918991
X...21470	1.04812	0.495364
X...24608	1.04737	0.250227
glycoursodeoxycholate	1.04726	0.478147
aconitate..cis.or.trans.	1.04701	0.60252
X1..1.enyl.palmitoyl..2.palmitoyl.GPC..P.16.0.16.0..	1.04585	0.879412
X...25343	1.045	0.915349
diphenhydramine(NA)	1.04491	2.08292
X...25790	1.04425	0.540193
gamma.glutamylcitrulline.	1.04301	0.811572
X...24556	1.04136	0.638379
cortisol	1.04004	0.375016
dimethylglycine	1.03984	0.544767
X...13835	1.03805	0.534939
N.acetylglucosaminylasparagine	1.0352	0.701024
taurine	1.03484	0.745325
X1..1.enyl.stearoyl..2.linoleoyl.GPE..P.18.0.18.2..	1.03455	0.773045

citalopram.propio.te.(NA)	1.03224	1.09445
X...23648	1.02983	0.344272
N.acetylcitrulline	1.02949	0.430564
gamma.CEHC.glucuronide.	1.02756	0.359413
X...21442	1.02471	0.226578
N.acetylasparagine	1.02426	0.736629
X2.hydroxyphenylacetate	1.02396	0.387142
C.glycosyltryptophan	1.01928	0.642851
glutamine.conjugate.of.C7H12O2.	1.01876	0.524385
X3.bromo.5.chloro.2.6.dihydroxybenzoic.acid.	1.01856	0.380064
X10.no.decenoate..19.1n9.	1.01836	1.04753
citrate	1.01799	0.696015
spermidine	1.01662	0.643619
S.methylcysteine	1.01469	0.947102
X2.ketocaprylate	1.01425	0.691519
X2.hydroxysebacate	1.01398	0.507922
indoleacetylglutamine	1.01391	0.555546
citalopram.escitalopram(NA)	1.01356	1.62554
X.16.or.17..methylstearate..a19.0.or.i19.0.	1.01327	0.467137
pimeloylcarnitine.3.methyladipoylcarnitine..C7.DC.	1.00813	0.204658
X4.vinylphenol.sulfate	1.00586	0.373611
X...21364	1.00458	0.427913
X1.methyl.5.imidazoleacetate	1.00326	0.459835
X2.hydroxyhippurate..salicylurate.	1.0012	0.283035
hexadecadienoate..16.2n6.	0.999559	1.55736
X1..1.enyl.palmitoyl..2.oleoyl.GPC..P.16.0.18.1..	0.999299	1.17757
N..2.furoyl.glycine	0.998967	0.44572
X...12729	0.997646	0.503171

glycine	0.997486	0.663389
androstenediol..3beta.17beta..monosulfate..1.	0.997449	0.70069
X...11858	0.995735	0.403248
N.methylhydroxyproline..	0.992385	0.677863
tyrosine	0.991072	0.336573
saccharin(NA)	0.989808	0.831763
bilirubin..E.E..	0.988005	0.508859
N.acetylmethionine	0.987049	0.892052
X...16576	0.98698	0.743623
sphinganine.1.phosphate	0.98657	0.434048
O.sulfo.L.tyrosine	0.98555	0.445685
dihomo.linoleate..20.2n6.	0.981431	1.45757
X1.stearoyl.2.oleoyl.GPS..18.0.18.1.	0.980394	0.734453
gamma.glutamylglutamine	0.979607	0.481361
linoleate..18.2n6.	0.97798	1.10901
X...15486	0.977927	0.933737
X...22771	0.976892	0.508202
taurodeoxycholate	0.976132	0.19516
eicosenoate..20.1.	0.974238	1.2034
X1.palmitoylglycerol..16.0.	0.972812	0.432413
taurocholate	0.97268	0.37271
tetradecanedioate..C14.DC.	0.97264	0.413767
X...25810	0.972508	0.661209
X...12847	0.971781	0.451363
isovalerylglycine	0.970836	0.742992
N.formylanthranilic.acid	0.970447	0.562746
sebacate..C10.DC.	0.968135	0.450041
X16.hydroxypalmitate	0.966396	0.565138

N.acetyl.isoputreanine	0.963231	0.847839
X...21310	0.962424	0.278196
cin.moylglycine	0.961421	0.851535
cis.4.decenoate..10.1n6..	0.960308	1.35256
X4.ethylcatechol.sulfate	0.957362	0.691053
thioproline	0.956125	0.72712
beta.citrylglutamate	0.955608	0.602925
ribulo.te.xylulo.te.lyxo.te.	0.954826	0.816081
X...24748	0.952572	0.620142
N.acetylglutamate	0.952123	0.581916
qui.te	0.950027	0.823839
X...24522	0.949458	0.37848
X1..1.enyl.palmitoyl..2.oleoyl.GPE..P.16.0.18.1..	0.948228	0.379011
X...21834	0.94798	0.462899
X...17367	0.94649	0.508406
X...18899	0.946392	0.656029
glycerophosphoinositol.	0.946363	0.616697
hydroxypioglitazone..M.IV.(NA)	0.943951	1.27056
ethyl.beta.glucopyranoside	0.943121	0.823021
taurochenodeoxycholate	0.94293	0.341674
X5.dodecenoate..12.1n7.	0.941056	1.056
X1.palmitoyl.2.linoleoyl.GPC..16.0.18.2.	0.939668	0.510278
stearido.te..18.4n3.	0.939394	0.351549
X...24334	0.935978	0.439818
X1.carboxyethyltyrosine	0.935104	0.356234
cholate	0.934635	0.569708
X2.methoxyhydroquinone.sulfate..1.	0.934612	0.238189
X3.hydroxybutyroylglycine..	0.933428	0.795384

S.1.pyrroline.5.carboxylate	0.932359	0.691505
X...25503(NA)	0.930918	0.838561
N6.N6.dimethyllysine	0.929833	0.433867
tetradecadienoate..14.2..	0.929322	1.42677
glycoursodeoxycholic.acid.sulfate..1.	0.929088	0.805185
N.methylproline	0.927193	0.756506
galacto.te	0.925728	0.484079
andro.steroid.monosulfate.C19H28O6S..1..	0.92376	0.821022
oleate.vacce.te..18.1.	0.923627	1.37871
X4.hydroxychlorothalonil	0.921902	0.564779
X3.hydroxybutyrate..BHBA.	0.921648	0.51991
linoleoylcarnitine..C18.2..	0.921583	0.450901
beta.hydroxyisovalerate	0.921026	0.53353
delta.CEHC	0.920184	0.437499
glycosyl.N.palmitoyl.sphingosine..d18.1.16.0.	0.916299	0.7664
X12.13.DiHOME	0.912747	0.387694
X3.ureidopropio.te	0.911368	0.447845
X2.aminooctanoate	0.908908	0.816227
glycosyl.ceramide..d18.2.24.1..d18.1.24.2..	0.908011	0.740133
perfluorooctanoate..PFOA.	0.906032	0.744998
dodecanedioate..C12.DC.	0.905819	0.519512
octadecanedioate..C18.DC.	0.905427	0.496777
X...16397	0.903586	0.97867
X...12411	0.902871	0.369623
betaine	0.902429	0.596555
X...24328	0.901105	0.684653
docosapentaenoate..n6.DPA..22.5n6.	0.900752	0.539417
X1.oleoyl.GPS..18.1.	0.900672	0.500315

X1.stearoyl.2.arachidonoyl.GPS..18.0.20.4.	0.900221	0.569421
gamma.glutamyl.epsilon.lysine	0.899505	0.518209
docosadienoate..22.2n6.	0.897631	1.23399
ferulic.acid.4.sulfate	0.89648	0.525493
gamma.glutamyl.2.aminobutyrate	0.895662	0.815149
fructosyllysine	0.895383	0.572878
topiramate(0.0376811)	0.89377	2.31684
metoprolol(0.124957)	0.89377	2.31684
ciprofloxacin(-0.270497)	0.89377	2.31684
saccharin(-0.0815355)	0.89377	2.31684
bradykinin..hydroxy.pro.3.(-0.646454)	0.89377	2.31684
bradykinin..des.arg.9.(-1.21706)	0.89377	2.31684
hydroxybupropion(0.619285)	0.89377	2.31684
pioglitazone(-0.164167)	0.89377	2.31684
Fibrinopeptide.A..phosphono.ser.3..(-0.545417)	0.89377	2.31684
HXGXA.(0.361861)	0.89377	2.31684
metoprolol.acid.metabolite.(-0.570399)	0.89377	2.31684
lidocaine(1.19304)	0.89377	2.31684
carbamazepine(-0.0692429)	0.89377	2.31684
Nimesulide(-0.121264)	0.89377	2.31684
nornicotine(-0.0105555)	0.89377	2.31684
carbamazepine.glucuronide.(0.00229736)	0.89377	2.31684
oxypurinol(0.0987588)	0.89377	2.31684
pseudoephedrine(0.357605)	0.89377	2.31684
quetiapine(-0.0792597)	0.89377	2.31684
doxycycline(-0.26579)	0.89377	2.31684
leucylglycine(0.116449)	0.89377	2.31684
mirtazapine(0.297806)	0.89377	2.31684

verapamil(0.654198)	0.89377	2.31684
hydroxypioglitazone..M.IV.(0.278919)	0.89377	2.31684
furosemide(-0.626798)	0.89377	2.31684
olmesartan(-0.621571)	0.89377	2.31684
daidzein.sulfate..2.(-0.294774)	0.89377	2.31684
alpha.hydroxymetoprolol(-0.134446)	0.89377	2.31684
rivaroxaban(-0.161578)	0.89377	2.31684
tadalafil(0.173701)	0.89377	2.31684
cetirizine(-0.0570996)	0.89377	2.31684
mycophenolic.acid.glucuronide(-0.0218367)	0.89377	2.31684
sulfamethoxazole(-0.0881757)	0.89377	2.31684
dexlansoprazole(0.525734)	0.89377	2.31684
metronidazole(-0.413699)	0.89377	2.31684
moxifloxacin(0.0724137)	0.89377	2.31684
fluco.zole(-0.106361)	0.89377	2.31684
THC.carboxylic.acid.glucuronide(-0.568102)	0.89377	2.31684
montelukast(-0.0573114)	0.89377	2.31684
midazolam(-1.54365)	0.89377	2.31684
tamoxifen(-1.17247)	0.89377	2.31684
Urolithin.B.glucuronide(0.0330479)	0.89377	2.31684
Gliclazide(1.68938)	0.89377	2.31684
X...12407(-0.448164)	0.89377	2.31684
X...16649(-0.237242)	0.89377	2.31684
X...17348(-0.181402)	0.89377	2.31684
X...21315(0.0980337)	0.89377	2.31684
X...21842(0.28277)	0.89377	2.31684
X...24576(0.341957)	0.89377	2.31684
X...25503(-0.147804)	0.89377	2.31684

X...25523(0.0615654)	0.89377	2.31684
caprylate..8.0.	0.89374	0.741972
X...12101	0.891608	0.867898
indolin.2.one	0.890285	0.344855
X1..1.enyl.palmitoyl..2.palmitoleoyl.GPC..P.16.0.16.1..	0.887809	0.873161
X...12680	0.884705	0.804626
asparagine	0.884633	0.452276
X...21829	0.883149	1.17562
X4.hydroxyhippurate	0.882294	0.371843
X3.methylglutaco.te	0.882016	0.889943
X...19141	0.879902	0.307459
X5.HETE	0.879766	1.0435
X...13007	0.879634	0.39974
X...17357	0.878035	0.678634
X6.bromotryptophan	0.877945	0.344745
palmitoleate..16.1n7.	0.875394	1.41128
glycohyocholate	0.874944	0.374331
gamma.tocopherol.beta.tocopherol	0.874349	0.639117
N6.methyllysine	0.87407	0.501158
orotidine	0.872981	0.393609
sphingosine	0.872682	1.06155
dehydroepiandrosterone.sulfate..DHEA.S.	0.872398	0.573126
X3.methylcytidine	0.872121	0.242547
N6.succinyladenosine	0.870545	0.733477
pyridoxate	0.870147	0.601305
gluco.te	0.869165	1.01599
pyrraline	0.868579	0.465818
X5alpha.androstan.3alpha.17alpha.diol.monosulfate	0.865413	0.63019

X3beta.7alpha.dihydroxy.5.cholestenoate	0.864064	0.50613
corticosterone	0.863326	0.340329
X...17185	0.86282	0.607155
taurodeoxycholic.acid.3.sulfate	0.860923	0.355983
X...15728	0.859021	0.387958
tadalafil(NA)	0.858455	1.15385
decadienedioic.acid..C10.2.DC...	0.858024	0.793415
X11.ketoetiocholanolone.glucuronide	0.855907	0.489368
methionine	0.855148	0.401861
X6.hydroxywarfarin(NA)	0.853845	1.65636
X...25520	0.85089	0.408085
imidazole.propio.te	0.847789	0.350688
X3.hydroxyhexanoate	0.847644	0.934738
histidine	0.845078	0.372042
X...24425	0.844257	0.666398
linole.te..alpha.or.gamma...18.3n3.or.6..	0.844077	1.21172
X3.hydroxyhippurate	0.844068	0.315886
X...21286	0.843474	0.718988
hydroquinone.sulfate	0.843411	0.372498
branched.chain..straight.chain..or.cyclopropyl.10.1.fatty.acid..1..	0.843246	0.854833
X4.chlorobenzoic.acid	0.842837	0.300633
androsterone.glucuronide	0.841311	0.358879
X...11407	0.840843	0.551597
mannitol.sorbitol	0.840764	0.7299
bradykinin(0.118316)	0.840606	2.35589
bradykinin..des.arg.9.(0.324544)	0.840606	2.35589
Fibrinopeptide.A.(-0.310882)	0.840606	2.35589

HXGXA.(-0.670017)	0.840606	2.35589
X2.methoxyacetaminophen.sulfate.(0.170924)	0.840606	2.35589
diphenhydramine(1.57516)	0.840606	2.35589
pseudoephedrine(0.640326)	0.840606	2.35589
X2.hydroxyibuprofen(-1.7464)	0.840606	2.35589
carboxyibuprofen(-2.62141)	0.840606	2.35589
X...17348(-1.72541)	0.840606	2.35589
X1..1.enyl.palmitoyl..2.linoleoyl.GPE..P.16.0.18.2..	0.838853	0.563997
N2.acetyllysine	0.836705	0.391991
X3.methyl.catechol.sulfate..1.	0.834236	0.402042
heptenedioate..C7.1.DC..	0.833547	0.615434
glutarylcarnitine..C5.DC.	0.833245	0.335291
X.14.or.15..methylpalmitate..a17.0.or.i17.0.	0.833127	0.944155
X3.methylxanthine	0.833054	0.304932
taurochenodeoxycholic.acid.3.sulfate	0.82769	0.40595
X2.aminobutyrate	0.826679	0.554552
X...11849	0.824886	0.431836
X3b.hydroxy.5.cholenoic.acid	0.822745	0.500277
X...18838	0.821844	0.485412
X3.phenylpropio.te..hydrocin.mate.	0.821149	0.680232
glycodeoxycholate	0.819924	0.351413
X...12216	0.819416	0.344862
X...21353	0.818157	1.44448
X...25419	0.818031	0.506461
X...11795	0.816315	0.648744
glyco.beta.muricholate..	0.815404	0.699034
heptanoate..7.0.	0.814621	0.692451
X1..1.enyl.palmitoyl..2.linoleoyl.GPC..P.16.0.18.2..	0.814312	1.28522

pentadecanoate..15.0.	0.814128	0.969789
orotate	0.812904	0.66883
fructose	0.811939	0.738125
desmethylcitalopram.(NA)	0.81141	1.14649
bradykinin..des.arg.9.(NA)	0.811024	0.707025
X.2.or.3..decenoate..10.1n7.or.n8.	0.810663	0.838598
X5.oxoproline	0.808341	0.685898
homovanillate..HVA.	0.808323	0.458619
cis.4.decenoylcarnitine..C10.1.	0.808136	1.13596
X3beta.hydroxy.5.cholestenoate	0.805963	0.326464
cystine	0.805878	0.41843
arginine	0.805704	0.633728
bradykinin(1.41932)	0.804415	2.25668
bradykinin..hydroxy.pro.3.(-0.124883)	0.804415	2.25668
bradykinin..des.arg.9.(0.903894)	0.804415	2.25668
Fibrinopeptide.A.(2.90452)	0.804415	2.25668
Fibrinopeptide.A..des.ala.1..(2.11233)	0.804415	2.25668
Fibrinopeptide.A..phosphono.ser.3..(1.01606)	0.804415	2.25668
HXGXA.(1.29815)	0.804415	2.25668
citalopram.escitalopram(-0.554081)	0.804415	2.25668
histidylalanine(-0.170788)	0.804415	2.25668
desmethylcitalopram.(-0.262014)	0.804415	2.25668
citalopram.propio.te.(-1.20631)	0.804415	2.25668
Fibrinopeptide.B..1.13...(1.74379)	0.804415	2.25668
X...25503(1.49669)	0.804415	2.25668
X2.hydroxybutyrate.2.hydroxyisobutyrate	0.803664	0.692983
X10.heptadecenoate..17.1n7.	0.802943	1.39742
X...12849	0.802487	0.335142

X...12283	0.801553	0.390534
X1.stearoyl.2.linoleoyl.GPI..18.0.18.2.	0.799485	0.668377
dihomo.linolenoyl.choline	0.798047	0.438063
X...11444	0.798038	0.708028
arachidonoylcholine	0.795629	0.829543
dihydroferulate	0.794246	0.391638
cysteine.glutathione.disulfide	0.79174	0.64192
glucuronide.of.C10H18O2..7..	0.789158	0.390442
X1.methylnicoti.mide	0.788902	0.831037
hypoxanthine	0.788812	0.693465
X...13728	0.788483	0.46764
N6.carboxymethyllysine	0.78769	0.412006
lidocaine(NA)	0.787001	0.608615
theanine	0.78621	0.462193
X...12026	0.785854	0.652839
X5.dodecenoylcarnitine..C12.1.	0.784009	0.443399
X...21607	0.783144	1.02726
cotinine	0.7823	0.486873
lanthionine	0.78201	0.573748
androsterone.sulfate	0.78185	0.453534
X...21851	0.780752	0.355312
glucuronide.of.C12H22O4..1..	0.779842	0.436311
X1.palmitoyl.GPA..16.0.	0.779122	0.732938
X...21383	0.778442	0.391197
butyrate.isobutyrate..4.0.	0.777505	0.333953
X...23780	0.776832	0.329468
X...24947	0.776351	0.503928
thymol.sulfate	0.776065	0.441909

X3.hydroxydecanoate	0.774868	1.46828
myristoleate..14.1n5.	0.773806	1.24855
X...21342	0.773394	0.514534
X3.hydroxyhippurate.sulfate	0.772016	0.253191
epiandrosterone.sulfate	0.771304	0.329944
X...14904	0.769792	0.352139
oxypurinol(-2.62417)	0.769513	1.82839
mycophenolic.acid(-1.98777)	0.769513	1.82839
meloxicam(-3.94248)	0.769513	1.82839
saccharin(-0.366292)	0.768034	2.40921
Fibrinopeptide.A..des.ala.1..(-0.672561)	0.768034	2.40921
X2.methoxyacetaminophen.sulfate.(-1.06857)	0.768034	2.40921
pantoprazole(-0.0230639)	0.768034	2.40921
X2.hydroxyibuprofen(-0.27194)	0.768034	2.40921
furosemide(0.211638)	0.768034	2.40921
o.hydroxyatorvastatin(0)	0.768034	2.40921
valsartan(0.0617534)	0.768034	2.40921
sitagliptin(-0.0646452)	0.768034	2.40921
phenylacetylmethionine(0.209369)	0.768034	2.40921
levetiracetam(2.01434)	0.768034	2.40921
THC.carboxylic.acid(-0.058795)	0.768034	2.40921
X...17348(1.39723)	0.768034	2.40921
X...21315(0.0138043)	0.768034	2.40921
X...21467	0.767259	0.673267
X16a.hydroxy.DHEA.3.sulfate	0.765783	0.715687
X...25420	0.764763	0.63072
X7.methylxanthine	0.759519	0.31757
N.acetylserine	0.759395	0.564559

X...11847	0.7577	0.148139
glutamine	0.757633	0.853051
X...21315(NA)	0.755207	0.69378
N.acetylalliin	0.755143	0.484612
resveratrol.disulfate..1..(NA)	0.753128	0.494281
X9.10.DiHOME	0.751573	0.571602
gamma.CEHC	0.750627	0.722777
creatine	0.750535	0.917614
benzoate	0.749867	0.812162
X...21821	0.749322	0.629686
daidzein.sulfate..2.(NA)	0.748305	0.773433
Fibrinopeptide.A..phosphono.ser.3..(NA)	0.746303	1.12331
X1ate	0.745535	0.55099
N.formylphenylalanine	0.742766	0.503696
adre.te..22.4n6.	0.74141	0.819292
X5alpha.androstan.3alpha.17beta.diol.17.glucuronide	0.741398	0.297212
X...25523(NA)	0.739148	0.773581
X...24669	0.737339	0.486881
X...12007	0.736693	0.515163
imidazole.lactate	0.736392	0.405952
X...18886	0.731209	0.385529
taurochole.te.sulfate.	0.727897	0.609352
decanoylcarnitine..C10.	0.727053	0.805169
cytidine	0.726648	0.977723
X5alpha.androstan.3alpha.17beta.diol.monosulfate..2.	0.72644	0.389184
ursodeoxycholate	0.725647	0.402756
X...24307	0.723019	0.501682
oleoylcholine	0.721715	0.61475

tryptophan.betaine	0.721134	0.166024
alpha.ketobutyrate	0.719923	0.432588
ectoine	0.718846	0.440342
HWESASXX.	0.718134	0.310456
taurolithocholate.3.sulfate	0.716993	0.461617
caprate..10.0.	0.716816	0.617075
glutamine.conjugate.of.C6H10O2..2..	0.716721	0.924226
heme	0.71612	0.307274
metoprolol.acid.metabolite.(-1.46837)	0.713258	1.85248
X2.methoxyacetaminophen.sulfate.(-2.1211)	0.713258	1.85248
diphenhydramine(-0.104916)	0.713258	1.85248
celecoxib(-0.96312)	0.713258	1.85248
valsartan(-3.37553)	0.713258	1.85248
diclofe.c(-0.616371)	0.713258	1.85248
X5alpha.androstan.3beta.17beta.diol.monosulfate..2.	0.711641	0.46882
gamma.glutamylthreonine	0.710721	0.391266
caproate..6.0.	0.710355	1.11899
S.allylcysteine	0.709827	0.550294
indole.3.carboxylate	0.708935	0.560901
phenylacetylmethionine(NA)	0.702581	0.531566
X3.hydroxyoctanoate	0.702357	1.5991
cysteine.sulfinic.acid	0.700075	0.409867
N.acetyl.beta.alanine	0.699774	0.447791
myristoleoylcarnitine..C14.1..	0.699222	0.892637
undecanedioate..C11.DC.	0.697119	0.727455
N.acetylhistidine	0.696669	0.387737
X5alpha.androstan.3beta.17alpha.diol.disulfate	0.696516	0.470923
X5alpha.androstan.3alpha.17beta.diol.monosulfate..1.	0.695377	0.432212

bradykinin(0.2714)	0.691713	1.62323
nornicotine(0.631059)	0.691713	1.62323
metoprolol.acid.metabolite.(0.74517)	0.690997	2.1146
lidocaine(0.270638)	0.690997	2.1146
X2.methoxyacetaminophen.sulfate.(0)	0.690997	2.1146
oxypurinol(1.83082)	0.690997	2.1146
atenolol(-0.245901)	0.690997	2.1146
ranitidine(0.26559)	0.690997	2.1146
pseudoephedrine(0.880664)	0.690997	2.1146
tramadol(-0.0213258)	0.690997	2.1146
O.desmethyltramadol.glucuronide(-0.24475)	0.690997	2.1146
alpha.hydroxymetoprolol(0.323893)	0.690997	2.1146
Urolithin.B.glucuronide(-0.679639)	0.690997	2.1146
Gliclazide(0)	0.690997	2.1146
X...12407(0)	0.690997	2.1146
palmitoylcholine	0.690963	0.805857
X...23644	0.688971	0.697234
nornicotine(NA)	0.688327	0.84646
norfluoxetine(NA)	0.685975	1.49628
glucuronide.of.piperine.metabolite.C17H21NO3..4..	0.685582	0.682202
phenylacetylglutamine	0.684546	0.935149
carboxyethyl.GABA	0.68364	0.46951
tyramine.O.sulfate	0.679378	0.58179
ribitol	0.678295	0.699389
N.acetyl.aspartyl.glutamate...AG.	0.675247	0.180847
O.desmethyltramadol.glucuronide(NA)	0.675037	1.2542
X...16938	0.674882	0.555538
sulfate.of.piperine.metabolite.C18H21NO3..3..	0.674171	0.34574

X2.3.dihydroxyisovalerate	0.671997	0.801624
dodecenedioate..C12.1.DC..	0.67194	0.747363
X...13507	0.667776	0.642026
X...24309	0.665059	0.281396
lactate	0.663064	0.475204
gamma.glutamylglycine	0.662757	0.517223
tetradecadienedioate..C14.2.DC..	0.661175	0.78427
salicylate	0.659937	0.271097
malate	0.659544	0.545172
X2..O.methylcytidine	0.655207	0.503239
N2.acetyl.N6.N6.dimethyllysine	0.65415	0.439083
X.N.1....N.8...acetylspermidine	0.653892	0.605065
Fibrinopeptide.A.(-0.271153)	0.653549	1.68345
Fibrinopeptide.A..des.ala.1..(0)	0.653549	1.68345
Fibrinopeptide.A..phosphono.ser.3..(-0.340661)	0.653549	1.68345
X2.methoxyacetaminophen.sulfate.(-2.63109)	0.653549	1.68345
bradykinin(0.0746435)	0.653013	1.63121
bradykinin..des.arg.9.(0.361304)	0.653013	1.63121
citalopram.escitalopram(-1.05814)	0.653013	1.63121
resveratrol.disulfate..1..(-0.50038)	0.653013	1.63121
citalopram.propio.te.(-0.668064)	0.653013	1.63121
X...16649(0.263056)	0.653013	1.63121
X...12812	0.652709	0.302889
threonine	0.651246	0.567158
propionylglycine	0.649514	0.473473
X2..deoxyuridine	0.649065	0.384351
azelate..C9.DC.	0.647685	0.471694
X2.aminophenol.sulfate	0.646214	0.351177

theobromine	0.646089	0.5344
fluoxetine(NA)	0.645445	1.27071
X...24243	0.643738	0.360054
X...21661	0.641858	0.413608
phenylacetate	0.637751	0.676869
glutarate..C5.DC.	0.637153	1.12689
uridine	0.636211	0.723086
X...17690	0.635584	0.519438
N.acetyltaurine	0.63556	0.424257
X5.6.dihydrothymine	0.635509	0.275281
X...18922	0.63538	0.996199
suberate..C8.DC.	0.633051	0.638188
beta.alanine	0.631582	0.342614
succi.te	0.630313	0.675989
X...12707	0.629694	0.419623
X...11470	0.627814	0.435981
X...16649(NA)	0.627273	0.642434
X...21796	0.625615	0.577426
succinimide	0.625103	0.328573
X8.hydroxyoctanoate	0.624917	0.528711
desmethylcitalopram.(0)	0.624411	1.48976
citalopram.propio.te.(0.149023)	0.624411	1.48976
indolepropio.te	0.622564	0.509247
histidylalanine(NA)	0.621519	0.551129
N.carbamoylalanine	0.62042	0.3022
tetrahydrocortisol.sulfate..1.	0.618771	0.538356
cholic.acid.glucuronide	0.616507	0.492361
X4.hydroxycoumarin	0.615989	0.774316

N.acetylaspartate...A.	0.612968	0.353112
acisoga	0.612892	0.763894
oxypurinol(NA)	0.612778	0.598741
bradykinin..hydroxy.pro.3.(NA)	0.612626	0.719738
N.N.dimethyl.5.aminovalerate	0.606552	0.421102
myristate..14.0.	0.60469	1.45596
vanillic.acid.glycine	0.603233	0.602359
octanoylcarnitine..C8.	0.602986	1.04503
X5.hydroxylysine	0.601499	0.382065
X1.linoleoyl.GPA..18.2..	0.599668	0.550328
X...12127	0.597165	0.407336
sucrose	0.595603	0.863789
X2.aminoheptanoate	0.594281	0.84023
X3.methylhistidine	0.592955	0.434146
glycolithocholate.sulfate.	0.592632	0.328924
deoxycholic.acid.12.sulfate.	0.591522	0.435539
beta.sitosterol	0.589743	0.554599
X...24747	0.588936	0.295939
nornicotine(0)	0.588727	1.43417
phenol.sulfate	0.586883	0.209516
Gliclazide(NA)	0.586797	0.430188
glycerol	0.585928	0.789096
X1.arachidonoyl.GPA..20.4.	0.584935	0.445318
phosphate	0.582845	0.407727
tramadol(NA)	0.580027	1.19393
methylsucci.te	0.571447	0.345111
thyroxine	0.569225	0.371633
glutamine.conjugate.of.C6H10O2..1..	0.567009	0.782645

nornicotine(0.457805)	0.566773	1.37144
doxycycline(0)	0.566773	1.37144
sulfamethoxazole(0.692997)	0.566773	1.37144
N4.acetylsulfamethoxazole.(0)	0.566773	1.37144
N4.acetylsulfamethoxazole.(NA)	0.566773	1.37144
N4.acetyl.5.hydroxysulfamethoxazole.(0)	0.566773	1.37144
X...21315(2.10457)	0.566773	1.37144
atenolol(NA)	0.566634	0.810402
X...18901	0.564143	0.325314
X9.hydroxystearate	0.563591	0.87711
phenolphthalein.beta.D.glucuronide(NA)	0.561879	0.513227
dihydrocaffeate.sulfate..2.	0.560601	0.313373
guanidinoacetate	0.559474	0.902393
N2.acetyl.N6.methyllysine	0.553317	0.595272
isoursodeoxycholate	0.551576	0.827066
metronidazole(0)	0.54864	0.34303
X2.methoxyacetaminophen.sulfate.(-2.11279)	0.547996	1.41074
oxypurinol(0.541277)	0.547996	1.41074
daidzein.sulfate..2.(1.22914)	0.547996	1.41074
tadalafil(0.00458945)	0.547996	1.41074
resveratrol.disulfate..1..(-0.173996)	0.547996	1.41074
X2.methoxyacetaminophen.sulfate.(-0.53273)	0.547308	1.37359
X2.hydroxyibuprofen(-0.239654)	0.547308	1.37359
carboxyibuprofen(-0.339677)	0.547308	1.37359
tadalafil(1.12142)	0.547308	1.37359
cetirizine(2.07481)	0.547308	1.37359
meloxicam(-0.434945)	0.547308	1.37359
Gliclazide(0.987643)	0.547308	1.37359

X...16649(-1.31677)	0.547308	1.37359
X...25523(0.312326)	0.547308	1.37359
THC.carboxylic.acid(NA)	0.546703	0.738056
X...11299	0.546335	0.872428
pantoate	0.545395	0.277998
X...12013	0.540517	0.429762
X...25422	0.539634	0.404797
dibutyl.sulfosucci.te	0.539253	0.319996
fumarate	0.538522	0.677118
.proxen(NA)	0.538437	0.486782
saccharin(-1.46968)	0.536324	1.22482
pioglitazone(-2.33408)	0.536324	1.22482
oxypurinol(0.442697)	0.536324	1.22482
hydroxypioglitazone..M.IV.(-2.02193)	0.536324	1.22482
X...21842(-0.187294)	0.536324	1.22482
X...17655	0.535089	0.428085
nornicotine(-0.0112632)	0.534915	1.23813
X...17348(-1.041)	0.534915	1.23813
X2.methoxyacetaminophen.sulfate.(-1.75215)	0.533376	1.42144
oxypurinol(-5.99146)	0.533376	1.42144
phenylacetylmethionine(-0.0678507)	0.533376	1.42144
dexlansoprazole(2.19185)	0.533376	1.42144
meloxicam(2.73568)	0.533376	1.42144
Gliclazide(0.799397)	0.533376	1.42144
X...16649(1.37985)	0.533376	1.42144
X...17348(-0.609174)	0.533376	1.42144
inosine	0.532545	0.463127
X...25265	0.532239	0.572202

X4.methylguaiacol.sulfate	0.531215	0.230149
bradykinin(-0.0215301)	0.527917	1.2762
bradykinin..hydroxy.pro.3.(-0.15059)	0.527917	1.2762
bradykinin..des.arg.9.(-0.20016)	0.527917	1.2762
X...16649(-0.380382)	0.527917	1.2762
X...21315(3.39949)	0.527917	1.2762
X...25523(0.753678)	0.527917	1.2762
X...15220	0.525867	0.211556
ranitidine(NA)	0.525142	0.527186
N.N.dimethylalanine	0.523191	0.731612
X...15461	0.522815	0.555941
metoprolol.acid.metabolite.(0.248031)	0.522649	1.51376
lidocaine(-0.199793)	0.522649	1.51376
oxypurinol(-0.245389)	0.522649	1.51376
atenolol(0.535499)	0.522649	1.51376
pseudoephedrine(-0.543005)	0.522649	1.51376
tramadol(-0.185246)	0.522649	1.51376
O.desmethyltramadol.glucuronide(-0.133531)	0.522649	1.51376
X...25503(0.719205)	0.522649	1.51376
X...12851	0.521879	0.395391
X...21842(2.09765)	0.52047	1.22684
X...25523(-1.78797)	0.52047	1.22684
phenolphthalein.beta.D.glucuronide(-5.9145)	0.520022	1.20382
Fibrinopeptide.A..des.ala.1..(0.000699755)	0.520022	1.20382
pseudoephedrine(-0.530688)	0.520022	1.20382
furosemide(-0.977103)	0.520022	1.20382
meloxicam(-0.0619818)	0.520022	1.20382
Fibrinopeptide.B..1.13...(-1.68255)	0.520022	1.20382

Gliclazide(0.722997)	0.520022	1.20382
gamma.carboxyglutamate	0.516849	0.362927
bradykinin(NA)	0.51584	0.747033
alpha.hydroxycaproate	0.51401	0.556722
saccharin(-0.598929)	0.512083	1.24481
metoprolol.acid.metabolite.(-0.0598562)	0.512083	1.24481
oxypurinol(5.17396)	0.512083	1.24481
atenolol(1.10333)	0.512083	1.24481
ranitidine(-1.18613)	0.512083	1.24481
mycophenolic.acid(0)	0.512083	1.24481
mycophenolic.acid.glucuronide(0)	0.512083	1.24481
resveratrol.disulfate..1..(0.313277)	0.512083	1.24481
ranitidine.N.oxide.(-0.760998)	0.512083	1.24481
X...17348(-0.629609)	0.512083	1.24481
pantoprazole(NA)	0.511724	0.79709
levetiracetam(0.0658814)	0.504567	1.19614
X...21842(-0.398688)	0.504567	1.19614
X...25523(-1.04412)	0.504567	1.19614
X2.methoxyacetaminophen.sulfate.(-0.919546)	0.499503	1.25566
X...21315(-0.370919)	0.499503	1.25566
palmitoleoylcarnitine..C16.1..	0.49946	0.372576
androstenediol..3beta.17beta..monosulfate..2.	0.499328	0.384976
daidzein.sulfate..2.(1.66744)	0.498014	1.20211
resveratrol.disulfate..1..(-0.621943)	0.498014	1.20211
meloxicam(0.32027)	0.498014	1.20211
Gliclazide(-0.232941)	0.498014	1.20211
X...12407(-0.12613)	0.498014	1.20211
X...16649(-0.263835)	0.498014	1.20211

X...17348(-0.434636)	0.498014	1.20211
X...21842(0.179233)	0.498014	1.20211
X...25503(-0.544727)	0.498014	1.20211
X...25523(-1.41511)	0.498014	1.20211
etiocholanolone.glucuronide	0.496311	0.381128
X...22520	0.494875	0.500784
metoprolol.acid.metabolite.(NA)	0.493589	1.27232
p.cresol.glucuronide.	0.490458	0.682161
THC.carboxylic.acid.glucuronide(0)	0.488733	0.534518
cytosine	0.488166	0.301062
glycolithocholate	0.487936	0.248904
X2.methoxyacetaminophen.sulfate.(-0.660874)	0.485991	1.11747
X...21842(0.284502)	0.485991	1.11747
.proxen(-0.213317)	0.485858	1.11689
X...17348(1.297)	0.485858	1.11689
levetiracetam(NA)	0.483431	0.329806
tadalafil(3.102)	0.483227	1.27721
THC.carboxylic.acid(-1.68902)	0.483227	1.27721
X...17348(-0.519698)	0.483227	1.27721
X...21842(-0.314163)	0.483227	1.27721
X...25503(0.0955829)	0.483227	1.27721
X...25267	0.482973	1.10997
X2.methoxyacetaminophen.sulfate.(-1.40039)	0.480239	1.13011
oxypurinol(0.0910283)	0.480239	1.13011
pantoprazole(4.25789)	0.480239	1.13011
dexlansoprazole(0.659435)	0.480239	1.13011
resveratrol.disulfate..1..(0.0580802)	0.480239	1.13011
ranitidine.N.oxide.(NA)	0.479729	1.18267

o.hydroxyatorvastatin(-0.844668)	0.479671	1.09591
X...16649(-0.304354)	0.479671	1.09591
X...17348(1.63575)	0.479671	1.09591
nornicotine(0.141413)	0.479209	1.19169
oxypurinol(-0.0719257)	0.479209	1.19169
atenolol(-0.749083)	0.479209	1.19169
X...17348(1.27787)	0.479209	1.19169
N.palmitoylserine	0.477395	0.335509
X...17348(NA)	0.477	0.400779
levetiracetam(-0.0487702)	0.475932	1.12714
resveratrol.disulfate..1..(0.594983)	0.473383	1.08862
X...25523(0.929404)	0.473383	1.08862
p.cresol.sulfate	0.473247	0.619203
X5.hydroxyhexanoate	0.472507	0.552011
X...17348(-0.516676)	0.471981	1.18471
X...25523(0.701958)	0.471981	1.18471
ethylmalo.te	0.47026	1.02427
mycophenolic.acid(NA)	0.470149	0.352892
N.acetyltheanine	0.469136	0.566609
metoprolol.acid.metabolite.(-0.395267)	0.467858	1.11627
lidocaine(1.55907)	0.467858	1.11627
X2.methoxyacetaminophen.sulfate.(0.0196065)	0.467858	1.11627
nornicotine(-0.395119)	0.467858	1.11627
THC.carboxylic.acid(0.627648)	0.467858	1.11627
meloxicam(0.423894)	0.467858	1.11627
Gliclazide(-1.4593)	0.467858	1.11627
X...21315(-1.39111)	0.467858	1.11627
X...12462	0.467152	0.702574

X2..O.methyluridine	0.466737	0.11279
X...11850	0.466066	0.615287
.proxen(0)	0.46504	0.640877
biliverdin	0.464109	0.862535
bradykinin(-0.0749391)	0.462281	1.07363
metronidazole(-0.237496)	0.462281	1.07363
resveratrol.disulfate..1..(1.6152)	0.462281	1.07363
X...21315(2.98019)	0.462281	1.07363
X2.methoxyacetaminophen.sulfate.(0.218493)	0.461083	1.13336
Nimesulide(-3.68888)	0.461083	1.13336
sitagliptin(-4.18646)	0.461083	1.13336
tadalafil(1.60609)	0.461083	1.13336
resveratrol.disulfate..1..(-0.574831)	0.461083	1.13336
gamma.glutamylalanine	0.460531	0.611474
hexanoylglutamine	0.460075	0.75842
X...21312	0.459583	0.397426
X3.hydroxy.3.methylglutarate	0.459232	0.256412
X...15664	0.457813	0.343878
bradykinin(0.692547)	0.457409	1.04743
bradykinin..hydroxy.pro.3.(-1.23203)	0.457409	1.04743
Fibrinopeptide.A.(-0.928363)	0.457409	1.04743
atenolol(-0.965744)	0.457409	1.04743
metoprolol.acid.metabolite.(0.0041912)	0.457173	1.10556
valsartan(-2.99174)	0.457173	1.10556
cetirizine(0)	0.457173	1.10556
meloxicam(-0.0436385)	0.457173	1.10556
linoleoylcholine.	0.45685	0.701912
X...21315(0)	0.455532	0.583436

X13.HODE...9.HODE	0.455083	1.10833
X...23974	0.454155	0.629011
X2.hydroxyibuprofen(NA)	0.453875	1.10672
bilirubin..Z.Z.	0.453593	0.677222
cysteine.s.sulfate	0.453422	0.247501
metoprolol.acid.metabolite.(-0.780668)	0.453237	1.04876
nornicotine(0.317872)	0.453237	1.04876
oxypurinol(4.91912)	0.453237	1.04876
phenylacetylmethionine(-0.508661)	0.453237	1.04876
Urolithin.B.glucuronide(-0.0657125)	0.453237	1.04876
Gliclazide(-3.54391)	0.453237	1.04876
X...21315(-1.94142)	0.453237	1.04876
X...21315(-2.85597)	0.452308	1.23496
X...25503(-0.714779)	0.452308	1.23496
X...25523(0.265666)	0.452308	1.23496
lidocaine(1.32447)	0.452158	1.06941
X2.methoxyacetaminophen.sulfate.(-0.357675)	0.452158	1.06941
pseudoephedrine(1.00063)	0.452158	1.06941
metoprolol.acid.metabolite.(0.354031)	0.450677	1.02341
lidocaine(-0.0499258)	0.450677	1.02341
atenolol(0.388183)	0.450677	1.02341
ranitidine(-0.0254204)	0.450677	1.02341
pseudoephedrine(-0.131933)	0.450677	1.02341
tramadol(-0.0235757)	0.450677	1.02341
O.desmethyltramadol.glucuronide(0.311521)	0.450677	1.02341
X...21315(-1.86562)	0.450677	1.02341
Gliclazide(-0.61804)	0.449591	1.05354
X...25957	0.447194	0.445296

carboxyibuprofen(-0.0851223)	0.446456	1.16387
N4.acetyl.5.hydroxysulfamethoxazole.(-0.4466)	0.446456	1.16387
carboxyibuprofen(NA)	0.444203	0.470625
Fibrinopeptide.A..des.ala.1..(1.10569)	0.443411	0.998068
Fibrinopeptide.A..phosphono.ser.3..(-0.50783)	0.443411	0.998068
warfarin(-0.346159)	0.443411	0.998068
daidzein.sulfate..2.(-0.226901)	0.443411	0.998068
X...21842(NA)	0.442744	0.359686
X3.aminoisobutyrate	0.442438	0.800355
saccharin(-1.43381)	0.442195	1.03333
phenylacetylmethionine(0.119382)	0.442195	1.03333
Urolithin.B.glucuronide(-1.59455)	0.442195	1.03333
Gliclazide(-0.710904)	0.442195	1.03333
X...21315(-0.38126)	0.442195	1.03333
laurate..12.0.	0.442085	0.808976
X...17348(-1.66178)	0.441765	1.0101
X...21315(0.163224)	0.441765	1.0101
Fibrinopeptide.A..des.ala.1..(-0.688757)	0.441441	1.08244
oxypurinol(-3.62684)	0.441441	1.08244
valsartan(-6.07485)	0.441441	1.08244
resveratrol.disulfate..1..(0.431133)	0.441441	1.08244
X...25523(-0.627734)	0.441441	1.08244
saccharin(-1.27726)	0.440614	1.10278
THC.carboxylic.acid(0.577512)	0.440614	1.10278
X2.methoxyacetaminophen.sulfate.(NA)	0.43806	0.580481
methyl.4.hydroxybenzoate.sulfate	0.434296	0.263642
levetiracetam(-0.317317)	0.433599	1.10192
phenylacetylglutamate	0.432078	0.63668

X1..1.enyl.palmitoyl..2.arachidonoyl.GPC..P.16.0.20.4..	0.431709	0.791064
metoprolol(0.168223)	0.431315	1.08881
lidocaine(-3.58994)	0.431315	1.08881
nornicotine(-1.01583)	0.431315	1.08881
THC.carboxylic.acid(0.757436)	0.431315	1.08881
THC.carboxylic.acid.glucuronide(-0.191282)	0.431315	1.08881
meloxicam(0.339681)	0.431315	1.08881
Gliclazide(0.34614)	0.431315	1.08881
meloxicam(-0.188018)	0.431198	1.01973
Gliclazide(0.700023)	0.431198	1.01973
nornicotine(-0.384487)	0.430878	1.00089
X2.hydroxyibuprofen(3.08413)	0.430878	1.00089
carboxyibuprofen(0.565939)	0.430878	1.00089
meloxicam(0.668393)	0.430878	1.00089
Gliclazide(-0.621943)	0.430878	1.00089
X...21315(-0.128402)	0.430878	1.00089
lithocholate.sulfate..1.	0.430801	0.437711
Fibrinopeptide.A.(-1.55211)	0.430221	1.01274
Fibrinopeptide.A..des.ala.1..(-0.69997)	0.430221	1.01274
Fibrinopeptide.A..phosphono.ser.3..(-1.554)	0.430221	1.01274
X...16649(-1.10352)	0.430221	1.01274
X...21315(0.31313)	0.429798	1.10442
sulfate.of.piperine.metabolite.C18H21NO3..1..	0.426662	0.211675
fluoxetine(0)	0.425705	0.97773
norfluoxetine(0)	0.425705	0.97773
daidzein.sulfate..2.(-0.934964)	0.425705	0.97773
Fibrinopeptide.B..1.13...(-0.853786)	0.425705	0.97773
X2.hydroxyibuprofen(-2.10455)	0.42322	1.13277

carboxyibuprofen(-3.34671)	0.42322	1.13277
daidzein.sulfate..2.(-1.16764)	0.42322	1.13277
diclofe.c(2.27744)	0.42322	1.13277
iminodiacetate..IDA.	0.421612	0.319411
valsartan(-3.15825)	0.421493	1.11228
levetiracetam(-0.422815)	0.421493	1.11228
X...16649(-1.97544)	0.421493	1.11228
X...25503(0.264516)	0.421493	1.11228
N4.acetyl.5.hydroxysulfamethoxazole.(NA)	0.420572	0.891213
X...23665	0.420534	0.522278
X...17137	0.417555	0.354585
bradykinin..hydroxy.pro.3.(0.569227)	0.417409	1.09467
oxypurinol(-0.0531898)	0.417409	1.09467
resveratrol.disulfate..1..(0.0415257)	0.417409	1.09467
montelukast(0.831168)	0.417409	1.09467
Gliclazide(0.0329511)	0.417409	1.09467
bilirubin..E.Z.or.Z.E..	0.414476	0.371797
Fibrinopeptide.A..des.ala.1..(-0.84397)	0.414242	0.970845
lidocaine(-1.63117)	0.414242	0.970845
phenylacetylmethionine(-0.444882)	0.414242	0.970845
levetiracetam(-0.665727)	0.414242	0.970845
daidzein.sulfate..2.(0.186728)	0.413939	0.98273
X...16649(-1.43339)	0.413939	0.98273
leucylglycine(-0.378774)	0.413326	1.0652
X...21842(-0.11149)	0.413326	1.0652
leucylglycine(0.583667)	0.412845	0.92112
X...17348(-0.535802)	0.412845	0.92112
Fibrinopeptide.A..des.ala.1..(-0.00883895)	0.412626	0.981983

metoprolol.acid.metabolite.(1.07367)	0.412626	0.981983
lidocaine(0.23127)	0.412626	0.981983
oxypurinol(2.06758)	0.412626	0.981983
atenolol(-0.530518)	0.412626	0.981983
pseudoephedrine(0.233965)	0.412626	0.981983
tramadol(-0.0134906)	0.412626	0.981983
O.desmethyltramadol.glucuronide(0.275888)	0.412626	0.981983
alpha.hydroxymetoprolol(0.422388)	0.412626	0.981983
phenylacetylmethionine(0.0331446)	0.412626	0.981983
Urolithin.B.glucuronide(0.637159)	0.412626	0.981983
Gliclazide(-1.57842)	0.412626	0.981983
X...21842(0.748676)	0.412626	0.981983
daidzein.sulfate..2.(0.501926)	0.411895	0.932818
X...16649(1.57082)	0.411895	0.932818
daidzein.sulfate..2.(1.59294)	0.411733	0.912507
X...16649(0.475799)	0.411733	0.912507
anthranilate	0.411538	0.29186
Fibrinopeptide.A..des.ala.1..(-1.24932)	0.410151	0.961569
trans.uroca.te	0.408466	0.58915
X...16649(-1.0567)	0.406458	0.977953
X...12544	0.404666	0.719569
dimethyl.sulfone	0.404062	0.321774
X...25523(0)	0.403781	0.769393
Fibrinopeptide.A..des.ala.1..(0.799442)	0.403217	0.942928
X2.methoxyacetaminophen.sulfate.(-0.578391)	0.403217	0.942928
oxypurinol(-0.682801)	0.403217	0.942928
oxypurinol(1.43723)	0.40313	0.93348
valsartan(-3.58272)	0.40313	0.93348

resveratrol.disulfate..1..(-0.150939)	0.40313	0.93348
meloxicam(1.79203)	0.40313	0.93348
Gliclazide(-0.911552)	0.40313	0.93348
X...17348(0.324978)	0.40313	0.93348
X2.methoxyacetaminophen.sulfate.(-0.00652122)	0.401368	0.929428
X...21315(-0.806764)	0.401368	0.929428
X...25503(-1.16507)	0.401368	0.929428
X2.methoxyacetaminophen.sulfate.(-0.201015)	0.401288	0.931987
bradykinin(-0.242836)	0.400801	0.944903
daidzein.sulfate..2.(-0.0465676)	0.400801	0.944903
X...16649(0.248889)	0.400801	0.944903
histidylalanine(-0.411282)	0.40021	0.930226
X...21315(-1.23169)	0.40021	0.930226
phenylacetylmethionine(-0.175783)	0.399023	0.951652
X...21315(0.912162)	0.399023	0.951652
X...21842(-0.674718)	0.399023	0.951652
X...25503(-1.54412)	0.399023	0.951652
resveratrol.disulfate..1..(0.00618086)	0.398437	0.987767
X...21842(-0.413245)	0.398437	0.987767
X...25523(2.32224)	0.398437	0.987767
daidzein.sulfate..2.(-0.63337)	0.398222	0.916738
X...21315(1.29102)	0.398222	0.916738
N.acetyl.cadaverine	0.394919	0.51778
X5.6.dihydrouracil	0.392022	0.533813
oxypurinol(-2.19463)	0.391151	0.901109
leucylglycine(-0.471124)	0.391151	0.901109
X...16649(0.851304)	0.391151	0.901109
X...17348(-1.78677)	0.391151	0.901109

X3.methoxycatechol.sulfate..1.	0.387825	0.482228
metoprolol.acid.metabolite.(0.889577)	0.387238	0.943996
lidocaine(0.209126)	0.387238	0.943996
X2.methoxyacetaminophen.sulfate.(-1.1498)	0.387238	0.943996
oxypurinol(2.42541)	0.387238	0.943996
atenolol(-0.246668)	0.387238	0.943996
pseudoephedrine(2.919)	0.387238	0.943996
warfarin(-6.57128)	0.387238	0.943996
tramadol(-0.0311811)	0.387238	0.943996
O.desmethyltramadol.glucuronide(0.215837)	0.387238	0.943996
alpha.hydroxymetoprolol(-0.159465)	0.387238	0.943996
cetirizine(-0.152452)	0.387238	0.943996
Fibrinopeptide.B..1.13...(-0.15923)	0.387238	0.943996
Gliclazide(-1.05125)	0.387238	0.943996
X...25523(1.99095)	0.387238	0.943996
bradykinin(0.540113)	0.386837	1.00885
bradykinin..hydroxy.pro.3.(-0.253474)	0.386837	1.00885
bradykinin..des.arg.9.(-0.573411)	0.386837	1.00885
Fibrinopeptide.A.(1.57543)	0.386837	1.00885
Fibrinopeptide.A..des.ala.1..(0.944645)	0.386837	1.00885
Fibrinopeptide.A..phosphono.ser.3..(-0.351119)	0.386837	1.00885
HXGXA.(-0.739191)	0.386837	1.00885
daidzein.sulfate..2.(-0.432168)	0.386837	1.00885
Fibrinopeptide.B..1.13...(-0.500545)	0.386837	1.00885
X...16649(1.29292)	0.386837	1.00885
X...17348(0.232856)	0.386837	1.00885
X4.hydroxynone.l	0.386649	0.284809
o.hydroxyatorvastatin(-0.416425)	0.384504	1.13761

X...16649(-1.52097)	0.384504	1.13761
X...21315(-0.837711)	0.384504	1.13761
X...21842(1.00737)	0.384504	1.13761
X...25503(-0.524925)	0.384504	1.13761
alliin	0.384277	0.443487
X...12798	0.384203	0.351973
atenolol(0.0706448)	0.383718	0.8893
X...21315(-1.27225)	0.383718	0.8893
metronidazole(-0.352114)	0.383466	0.891576
X2.methoxyacetaminophen.sulfate.(0.681429)	0.382026	0.902375
X2.methoxyacetaminophen.sulfate.(0.455905)	0.380947	0.955274
Gliclazide(-0.948298)	0.380947	0.955274
celecoxib(-1.74813)	0.379541	0.862961
sitagliptin(-3.21888)	0.379541	0.862961
X...25503(-1.28772)	0.379541	0.862961
X...25523(0.126192)	0.379541	0.862961
Fibrinopeptide.B..1.13...(NA)	0.379274	1.06406
X...11483	0.378454	0.515577
X...17685	0.375268	0.443867
bradykinin(0.276722)	0.374339	0.966016
bradykinin..hydroxy.pro.3.(-0.65778)	0.374339	0.966016
bradykinin..des.arg.9.(-0.000700245)	0.374339	0.966016
Fibrinopeptide.A.(1.26022)	0.374339	0.966016
Fibrinopeptide.A..des.ala.1..(0.961149)	0.374339	0.966016
Fibrinopeptide.A..phosphono.ser.3..(-0.538368)	0.374339	0.966016
HXGXA.(-0.6757)	0.374339	0.966016
X2.methoxyacetaminophen.sulfate.(-2.05494)	0.374339	0.966016
daidzein.sulfate..2.(1.65372)	0.374339	0.966016

resveratrol.disulfate..1..(0.0691529)	0.374339	0.966016
Fibrinopeptide.B..1.13...(-0.350409)	0.374339	0.966016
Gliclazide(0.729142)	0.374339	0.966016
X...16649(3.21725)	0.374339	0.966016
X...21842(-0.0648586)	0.374339	0.966016
lidocaine(0.202859)	0.374131	1.05115
phenylacetylmethionine(-0.502692)	0.374131	1.05115
X...21842(0.810263)	0.374131	1.05115
X...25523(-0.654696)	0.374131	1.05115
metoprolol(0.0282959)	0.373142	1.02269
X...25503(-0.684187)	0.372431	0.88346
saccharin(-1.96969)	0.371702	1.0575
X...21842(-0.457601)	0.371702	1.0575
X...25523(-0.0430119)	0.371702	1.0575
X3..3.hydroxyphenyl.propio.te	0.370703	0.200837
lidocaine(0.416207)	0.370452	0.883431
diclofe.c(-0.0335568)	0.370452	0.883431
X...17348(-1.4435)	0.370452	0.883431
bradykinin(-1.52005)	0.36921	0.883783
X...16649(0.351572)	0.36921	0.883783
X...25503(0.40139)	0.368791	0.83113
X...11843	0.368549	0.50371
Fibrinopeptide.A.(4.39499)	0.365346	0.851205
Fibrinopeptide.A..des.ala.1..(5.51723)	0.365346	0.851205
Fibrinopeptide.A..phosphono.ser.3..(0)	0.365346	0.851205
X2.hydroxyibuprofen(-0.0483503)	0.365346	0.851205
Fibrinopeptide.B..1.13...(4.14934)	0.365346	0.851205
X...21315(-0.689554)	0.365346	0.851205

X...25503(1.46168)	0.365346	0.851205
X...25523(-0.266965)	0.365346	0.851205
metoprolol.acid.metabolite.(-2.12026)	0.365236	0.853452
Fibrinopeptide.A..des.ala.1..(-0.421442)	0.365143	0.969117
X2.hydroxyibuprofen(-0.116534)	0.365143	0.969117
X...25503(-0.345452)	0.365143	0.969117
oxypurinol(1.07186)	0.363575	0.978696
X...16649(-1.10533)	0.363575	0.978696
X...25503(-0.128174)	0.363575	0.978696
Urolithin.B.glucuronide(-0.693947)	0.363463	0.877752
X...22776	0.362878	0.407144
oxypurinol(-0.195528)	0.361997	0.865405
X...25503(0.540462)	0.361997	0.865405
valsartan(-4.26158)	0.361704	0.860619
X...21315(0.562583)	0.361704	0.860619
pseudoephedrine(-2.62417)	0.359209	0.831112
Gliclazide(-1.27011)	0.359209	0.831112
bradykinin(-1.30048)	0.355988	1.0103
Fibrinopeptide.A.(0.0574195)	0.355988	1.0103
Fibrinopeptide.A..des.ala.1..(0.629248)	0.355988	1.0103
leucylglycine(-0.427557)	0.355988	1.0103
Fibrinopeptide.B..1.13...(-0.672561)	0.355988	1.0103
phenolphthalein.beta.D.glucuronide(0)	0.354289	0.788638
tadalafil(-3.3326)	0.35405	0.910511
mannose	0.353646	0.478117
resveratrol.disulfate..1..(-0.56247)	0.351869	0.821077
Gliclazide(-1.2365)	0.351869	0.821077
Fibrinopeptide.A..des.ala.1..(-0.597473)	0.35181	0.793875

X...21315(0.230318)	0.35181	0.793875
tauro.beta.muricholate	0.348473	0.425833
X...16649(-0.767224)	0.34695	0.830162
X...21842(0.46656)	0.34695	0.830162
lidocaine(-1.82822)	0.346761	0.864939
phenylacetylmethionine(0.58762)	0.346761	0.864939
bradykinin(-0.406315)	0.346492	0.797321
bradykinin..des.arg.9.(-3.0139)	0.346492	0.797321
X2.methoxyacetaminophen.sulfate.(1.18384)	0.346492	0.797321
Gliclazide(0.957394)	0.346492	0.797321
meloxicam(NA)	0.346407	0.451711
saccharin(-2.07147)	0.345629	0.787677
Gliclazide(-1.28989)	0.345629	0.787677
diclofe.c(-0.368169)	0.344514	0.992176
oxypurinol(-1.65653)	0.343925	0.843927
bradykinin(-2.78062)	0.343797	0.810685
Fibrinopeptide.A.(-2.27497)	0.343797	0.810685
Fibrinopeptide.A..des.ala.1..(-1.82822)	0.343797	0.810685
daidzein.sulfate..2.(0.855266)	0.343797	0.810685
Urolithin.B.glucuronide(-0.522392)	0.343797	0.810685
Gliclazide(-0.771758)	0.343797	0.810685
X...21842(-0.214679)	0.343797	0.810685
X...25523(-0.627172)	0.343797	0.810685
Urolithin.B.glucuronide(-1.08738)	0.343172	0.768107
X...21315(-1.35209)	0.343172	0.768107
bradykinin(0.577456)	0.341451	0.839996
bradykinin..hydroxy.pro.3.(0.151862)	0.341451	0.839996
bradykinin..des.arg.9.(-0.479973)	0.341451	0.839996

HXGXA.(-1.3123)	0.341451	0.839996
X2.methoxyacetaminophen.sulfate.(1.25416)	0.341451	0.839996
tadalafil(0.390893)	0.339135	0.858225
X...17348(-0.757152)	0.339135	0.858225
X...11852	0.337439	0.324214
X...13658	0.337429	0.586913
X2.methoxyacetaminophen.sulfate.(-1.98777)	0.337025	0.793817
THC.carboxylic.acid(-2.62693)	0.337025	0.793817
X...17348(-0.0991576)	0.337025	0.793817
Fibrinopeptide.A..des.ala.1..(1.22454)	0.336875	0.778892
Fibrinopeptide.A.(0)	0.336446	0.65093
levetiracetam(-0.455391)	0.336387	0.792746
X...21315(3.67334)	0.336387	0.792746
X...21842(-0.410377)	0.336387	0.792746
Fibrinopeptide.A.(-0.86893)	0.336253	0.730741
oxypurinol(-0.364275)	0.336253	0.730741
bradykinin..hydroxy.pro.3.(0)	0.335834	0.649119
X...25503(0.911318)	0.333796	0.787305
nornicotine(-0.391562)	0.333278	0.73568
X...21842(1.33597)	0.333013	0.799056
X...25523(1.1709)	0.333013	0.799056
acetylcarnitine..C2.	0.331965	0.675708
sulfate.of.piperine.metabolite.C16H19NO3..2..	0.331534	0.477258
leucylglycine(0)	0.330944	0.473972
saccharin(-2.43383)	0.329399	0.793292
phenylacetylmethionine(-0.540598)	0.329399	0.793292
Urolithin.B.glucuronide(-0.182002)	0.329399	0.793292
X...16649(0.347624)	0.329399	0.793292

X...21315(1.32909)	0.329399	0.793292
X...25503(0.0369104)	0.329399	0.793292
oxypurinol(-2.56916)	0.328777	0.825722
meloxicam(0.558072)	0.328777	0.825722
Gliclazide(-0.646645)	0.328777	0.825722
X...16649(1.70163)	0.328777	0.825722
oxypurinol(-2.2966)	0.327386	0.763535
phenylacetylmethionine(-0.760142)	0.327386	0.763535
X...16649(-0.972068)	0.327386	0.763535
pantoprazole(-3.07261)	0.32672	1.00375
warfarin(2.54657)	0.32672	1.00375
furosemide(-1.92964)	0.32672	1.00375
valsartan(-1.27368)	0.32672	1.00375
phenylacetylmethionine(-0.159465)	0.32672	1.00375
resveratrol.disulfate..1..(-0.100594)	0.32672	1.00375
X...21315(-0.32186)	0.32672	1.00375
X4.methylcatechol.sulfate	0.326171	0.236512
Fibrinopeptide.A.(-1.0128)	0.324813	0.783109
Fibrinopeptide.A..des.ala.1..(-0.182482)	0.324813	0.783109
diclofe.c(NA)	0.322852	0.347948
X...22802	0.322542	0.221429
Gliclazide(-1.5828)	0.32236	0.755351
nornicotine(-0.328226)	0.319809	0.756665
oxypurinol(0.352486)	0.319809	0.756665
valsartan(-0.965218)	0.319809	0.756665
X...21315(-0.266443)	0.319809	0.756665
X2.methoxyacetaminophen.sulfate.(0.031111)	0.318857	0.731906
lidocaine(-2.46158)	0.317278	0.82502

oxypurinol(-1.78916)	0.317278	0.82502
X...21315(-0.531198)	0.317278	0.82502
bradykinin(-0.0900341)	0.316439	0.753624
bradykinin..hydroxy.pro.3.(0.316124)	0.316439	0.753624
bradykinin..des.arg.9.(-1.36728)	0.316439	0.753624
Fibrinopeptide.A.(-1.49343)	0.316439	0.753624
Fibrinopeptide.A..des.ala.1..(-0.84072)	0.316439	0.753624
Fibrinopeptide.B..1.13...(-1.32351)	0.316439	0.753624
pantoprazole(-0.661067)	0.316047	0.774708
ketopioglitazone(0)	0.316047	0.774708
sitagliptin(-3.61192)	0.316047	0.774708
resveratrol.disulfate..1..(-0.65143)	0.316047	0.774708
bradykinin(0)	0.315502	0.511033
fluoxetine(1.82106)	0.314014	0.778755
norfluoxetine(1.97233)	0.314014	0.778755
warfarin(-7.6009)	0.314014	0.778755
X...16649(0.0517382)	0.314014	0.778755
X...21315(-0.381407)	0.314014	0.778755
X...25433	0.313901	0.458972
ranitidine(-1.22861)	0.313776	0.742554
leucylglycine(-0.312795)	0.313776	0.742554
X...17348(3.10147)	0.313776	0.742554
leucylglycine(-0.478358)	0.313723	0.736044
X2.methoxyacetaminophen.sulfate.(1.57296)	0.313043	0.724025
metoprolol(-0.0175532)	0.31273	0.764185
Fibrinopeptide.A..des.ala.1..(-0.305303)	0.31273	0.764185
X...21842(0.036814)	0.31273	0.764185
X...16649(0)	0.312521	0.174333

pantoprazole(0.0427721)	0.311599	0.813588
X2.hydroxyibuprofen(0.176387)	0.311599	0.813588
X...16649(0.645951)	0.311599	0.813588
sulfate.of.piperine.metabolite.C16H19NO3..3..	0.310239	0.376965
X...24576(-2.04949)	0.308978	0.728731
bradykinin(-0.961288)	0.307533	0.910385
HXGXA.(-0.114401)	0.307533	0.910385
leucylglycine(-0.0497156)	0.307533	0.910385
Fibrinopeptide.B..1.13...(-1.09005)	0.307533	0.910385
Gliclazide(-3.21389)	0.307533	0.910385
X...17348(-1.22418)	0.307533	0.910385
X...21315(-1.14917)	0.307533	0.910385
X...25503(-1.63322)	0.307533	0.910385
X...25523(-1.39191)	0.307533	0.910385
Gliclazide(0.65097)	0.304884	0.748265
X...25503(0.511206)	0.304884	0.748265
X2.methoxyacetaminophen.sulfate.(0.339468)	0.304832	0.722946
X...16649(0.38859)	0.304832	0.722946
ketopioglitazone(NA)	0.303896	0.445
leucylglycine(0.418447)	0.303838	0.920445
phenylacetylmethionine(0)	0.303838	0.920445
X...16649(0.349177)	0.303838	0.920445
X...12407(1.09788)	0.303765	0.732073
oxypurinol(0.386486)	0.302032	0.751372
metronidazole(-2.44993)	0.302032	0.751372
X3.hydroxysebacate	0.30103	0.453033
warfarin(-0.29303)	0.298139	0.719741
Gliclazide(-2.94314)	0.298139	0.719741

warfarin(-5.09947)	0.297406	0.702449
furosemide(-1.4662)	0.297406	0.702449
X...21842(-1.04897)	0.297406	0.702449
X...25523(-0.529159)	0.297406	0.702449
nornicotine(0.964967)	0.296495	0.706164
atenolol(-1.08945)	0.296495	0.706164
pantoprazole(-0.343054)	0.296058	0.82245
metronidazole(0.686676)	0.296058	0.82245
resveratrol.disulfate..1..(-0.764858)	0.296058	0.82245
Fibrinopeptide.A.(NA)	0.295518	0.449082
warfarin(NA)	0.294104	0.481775
X2.piperidinone	0.292004	0.626663
piperine	0.290294	0.387124
X...17348(0.967402)	0.289252	0.680955
fluoxetine(-3.2264)	0.286075	0.668274
nornicotine(-0.083599)	0.286075	0.668274
X...16649(0.252003)	0.286075	0.668274
oxypurinol(2.23972)	0.283073	0.833758
Gliclazide(-0.291556)	0.283073	0.833758
lidocaine(-0.940583)	0.281945	0.705968
X...16649(-0.277468)	0.281945	0.705968
lidocaine(-3.2114)	0.280922	0.658352
meloxicam(-3.4389)	0.280922	0.658352
Gliclazide(-2.96037)	0.280922	0.658352
X...21315(2.95435)	0.280922	0.658352
valsartan(NA)	0.280607	0.580812
resveratrol.disulfate..1..(0.179902)	0.276426	0.677707
Gliclazide(-2.28475)	0.276201	0.714871

X...17348(1.16159)	0.276201	0.714871
celecoxib(NA)	0.27496	0.437684
lidocaine(-0.412037)	0.272334	0.661872
X2.hydroxyibuprofen(-3.89222)	0.272334	0.661872
X...16649(-0.956592)	0.272025	0.650395
fluoxetine(-1.67131)	0.27188	0.732341
norfluoxetine(0.125575)	0.27188	0.732341
daidzein.sulfate..2.(-0.650663)	0.27188	0.732341
Gliclazide(-0.72237)	0.27188	0.732341
X...16649(0.574533)	0.27188	0.732341
X...21842(0.273152)	0.27188	0.732341
X...21842(0)	0.270414	0.421069
X...17348(0.925885)	0.270062	0.618752
Urolithin.B.glucuronide(-1.17733)	0.268464	0.623029
X...17348(-2.33511)	0.268177	0.645062
X...21315(-0.911303)	0.268177	0.645062
bradykinin(0.357325)	0.266831	0.681644
X...21842(0.77242)	0.266831	0.681644
metronidazole(2.43646)	0.265472	0.592535
sitagliptin(NA)	0.263518	0.790887
X...16649(0.0868197)	0.263288	0.676161
bradykinin(-0.619153)	0.262195	0.719723
bradykinin..hydroxy.pro.3.(-0.784167)	0.262195	0.719723
Fibrinopeptide.A.(-1.90784)	0.262195	0.719723
Fibrinopeptide.A..des.ala.1..(-1.15423)	0.262195	0.719723
phenylacetylmethionine(0.476296)	0.262195	0.719723
Urolithin.B.glucuronide(-0.237877)	0.262195	0.719723
Gliclazide(-0.407517)	0.262195	0.719723

X...21842(-0.785043)	0.262195	0.719723
X...25503(2.48059)	0.262195	0.719723
X...23997	0.26218	0.382135
X2.methoxyacetaminophen.sulfate.(-0.517179)	0.260903	0.614048
phenylacetylmethionine(0.113775)	0.260903	0.614048
valsartan(-2.69267)	0.260067	0.628581
leucylglycine(0.168476)	0.259494	0.623844
X...25503(-1.04441)	0.259494	0.623844
metoprolol.acid.metabolite.(-1.90987)	0.258177	0.641822
oxypurinol(-0.203341)	0.258177	0.641822
atenolol(0.107778)	0.258177	0.641822
X...16649(0.176974)	0.258177	0.641822
metoprolol.acid.metabolite.(0.486984)	0.257681	0.653341
lidocaine(0.005286)	0.257681	0.653341
atenolol(0.30476)	0.257681	0.653341
pseudoephedrine(-0.140527)	0.257681	0.653341
tramadol(0.422978)	0.257681	0.653341
O.desmethyltramadol.glucuronide(0.354031)	0.257681	0.653341
alpha.hydroxymetoprolol(0)	0.257681	0.653341
Urolithin.B.glucuronide(-0.908571)	0.257681	0.653341
X2.methoxyacetaminophen.sulfate.(1.35043)	0.256009	0.613031
Gliclazide(-3.18689)	0.255848	0.612474
X...16649(-1.54787)	0.255848	0.612474
X...16124	0.254995	0.157014
X2.methoxyacetaminophen.sulfate.(-3.41428)	0.253387	0.604267
histidylalanine(-0.231554)	0.253387	0.604267
warfarin(1.92343)	0.253387	0.604267
pantoprazole(-3.4546)	0.252294	0.578362

metronidazole(-4.35053)	0.252294	0.578362
phenylacetylmethionine(-0.589508)	0.252094	0.664806
X...21315(-1.18417)	0.252094	0.664806
X...25503(1.83133)	0.252094	0.664806
daidzein.sulfate..2.(2.83547)	0.251444	0.619745
X...16649(-0.220148)	0.251444	0.619745
X2.methoxyacetaminophen.sulfate.(1.43423)	0.251316	0.599077
metoprolol.acid.metabolite.(0.121775)	0.249377	0.71808
pantoprazole(-3.55435)	0.249377	0.71808
alpha.hydroxymetoprolol(-3.41125)	0.249377	0.71808
Gliclazide(0.941139)	0.249377	0.71808
nornicotine(0.0581746)	0.249085	0.603786
metronidazole(-1.01971)	0.249085	0.603786
Gliclazide(-0.0544562)	0.249085	0.603786
X...21842(0.248109)	0.247057	0.58004
X...25523(-0.951659)	0.247057	0.58004
X...21315(-1.15138)	0.247032	0.615297
nornicotine(0.327936)	0.245628	0.572502
saccharin(-1.72149)	0.244677	0.727952
X2.methoxyacetaminophen.sulfate.(-2.28573)	0.244677	0.727952
daidzein.sulfate..2.(0.113507)	0.244677	0.727952
X...17348(-1.22112)	0.244677	0.727952
X...21315(2.52158)	0.244677	0.727952
furosemide(-1.87732)	0.243916	0.603678
valsartan(-0.806316)	0.243916	0.603678
X...25503(2.41205)	0.243916	0.603678
carboxyibuprofen(-1.3579)	0.243296	0.609668
meloxicam(-3.81218)	0.243296	0.609668

X4.methylbenzenesulfo.te	0.242614	0.392298
warfarin(-0.577856)	0.241944	0.61322
valsartan(-0.00893984)	0.241944	0.61322
phenylacetylmethionine(-0.585908)	0.241944	0.61322
levetiracetam(-0.0500309)	0.241944	0.61322
resveratrol.disulfate..1..(-0.542316)	0.241944	0.61322
X...16649(-1.05613)	0.241944	0.61322
X...17348(-0.183682)	0.241944	0.61322
X...21842(-0.205795)	0.241944	0.61322
Fibrinopeptide.A..des.ala.1..(NA)	0.240826	0.422595
metoprolol.acid.metabolite.(0)	0.239317	0.637124
ranitidine(0.684258)	0.239317	0.637124
alpha.hydroxymetoprolol(-2.41912)	0.239317	0.637124
ranitidine.N.oxide.(0.381582)	0.239317	0.637124
X5.hydroxyindole.sulfate	0.238539	0.262011
X4.imidazoleacetate	0.236035	0.478104
bradykinin(-0.773057)	0.235818	0.617701
bradykinin..hydroxy.pro.3.(-0.517011)	0.235818	0.617701
uracil	0.233727	0.26893
diclofe.c(-0.0327298)	0.232224	0.529559
resveratrol.disulfate..1..(0.800251)	0.232224	0.529559
resveratrol.disulfate..1..(-0.0905814)	0.230738	0.624179
X2.methoxyacetaminophen.sulfate.(-0.216913)	0.230713	0.56333
oxypurinol(-0.245901)	0.228441	0.560159
warfarin(-0.881648)	0.228441	0.560159
furosemide(2.12479)	0.228441	0.560159
X6.hydroxywarfarin(0)	0.228441	0.560159
X2.methoxyacetaminophen.sulfate.(0.795388)	0.226399	0.553237

Fibrinopeptide.A..des.ala.1..(0.501078)	0.225767	0.545807
X...16649(-1.45758)	0.225767	0.545807
nornicotine(-0.787238)	0.22111	0.523348
X...24576(-2.67801)	0.22111	0.523348
levetiracetam(-0.12579)	0.220726	0.557842
lidocaine(2.02543)	0.218672	0.5476
Gliclazide(1.01693)	0.218672	0.5476
bradykinin(-0.979497)	0.218499	0.559384
venlafaxine(0)	0.218499	0.559384
venlafaxine(NA)	0.218499	0.559384
X2.hydroxyibuprofen(-0.266834)	0.218499	0.559384
diclofe.c(-0.483535)	0.218499	0.559384
oxypurinol(0)	0.216774	0.527192
Gliclazide(-0.788557)	0.216774	0.527192
X2.methoxyacetaminophen.sulfate.(-1.81462)	0.215122	0.532567
pseudoephedrine(2.11676)	0.215122	0.532567
X...25503(-0.390675)	0.215122	0.532567
oxypurinol(0.524077)	0.210718	0.555378
pioglitazone(-4.15409)	0.208214	0.516509
ketopioglitazone(1.58176)	0.208214	0.516509
hydroxypioglitazone..M.IV.(-2.3838)	0.208214	0.516509
resveratrol.disulfate..1..(-1.89845)	0.208214	0.516509
X...12407(0.0362355)	0.206701	0.457302
saccharin(-1.28049)	0.202904	0.510608
X2.methoxyacetaminophen.sulfate.(0.554517)	0.202904	0.510608
X...16649(-0.371209)	0.202904	0.510608
X2.methoxyacetaminophen.sulfate.(-1.24271)	0.20202	0.543042
HXGXA.(-0.94572)	0.201466	0.505047

oxypurinol(5.31488)	0.201466	0.505047
leucylglycine(-0.485483)	0.201466	0.505047
Gliclazide(0.537838)	0.201466	0.505047
X...21842(2.44533)	0.201466	0.505047
lidocaine(0.484153)	0.1968	0.497344
Gliclazide(-0.795623)	0.1968	0.497344
oxypurinol(-0.147688)	0.19579	0.487823
Gliclazide(-1.09333)	0.195779	0.493262
X...21315(-0.288482)	0.195779	0.493262
X2.methoxyacetaminophen.sulfate.(-1.21638)	0.195264	0.501061
oxypurinol(-3.33822)	0.195264	0.501061
resveratrol.disulfate..1..(0.236415)	0.195264	0.501061
Gliclazide(-0.583755)	0.195264	0.501061
X...21842(-0.721752)	0.195264	0.501061
X...17348(-0.992362)	0.194175	0.523452
X...16649(0.974371)	0.193068	0.474135
X...17348(-1.58719)	0.193068	0.474135
lidocaine(-4.01184)	0.19267	0.453148
nornicotine(-0.59602)	0.190906	0.44196
oxypurinol(0.81731)	0.190906	0.44196
resveratrol.disulfate..1..(-0.0407178)	0.19082	0.5367
lidocaine(-0.575542)	0.189765	0.56915
tramadol(-5.05146)	0.189765	0.56915
X...17348(-0.148152)	0.189765	0.56915
Fibrinopeptide.A..des.ala.1..(-0.749083)	0.189542	0.453701
oxypurinol(-1.32238)	0.184983	0.418645
carboxyibuprofen(-0.476907)	0.184983	0.418645
X2.methoxyacetaminophen.sulfate.(-0.993712)	0.174192	0.445973

oxypurinol(3.01618)	0.173484	0.422985
diclofe.c(-0.829425)	0.173484	0.422985
metoprolol(-0.21406)	0.172975	0.469801
carboxyibuprofen(-3.21888)	0.172975	0.469801
X...25523(-1.36806)	0.172975	0.469801
X...25503(-2.15935)	0.16879	0.415194
phenylacetylmethionine(-0.447069)	0.166281	0.397345
levetiracetam(-0.408269)	0.166281	0.397345
phenylacetylmethionine(-0.533924)	0.16559	0.398307
X...21315(-0.569868)	0.16397	0.415089
X...17348(-2.3279)	0.161308	0.423575
X2.methoxyacetaminophen.sulfate.(-2.94314)	0.160212	0.527591
oxypurinol(-1.66231)	0.1567	0.407149
Gliclazide(1.59148)	0.1567	0.407149
X...25523(0.267428)	0.1567	0.407149
pioglitazone(0.435153)	0.155292	0.40362
metoprolol.acid.metabolite.(2.03332)	0.155292	0.40362
